# Engineering Approaches to Modify Immunomodulatory Functions of Mesenchymal Stromal Cells (MSCs): Tissue Regeneration and Clinical Application

**DOI:** 10.1002/advs.202522601

**Published:** 2026-03-23

**Authors:** Sichen Yang, Kejia Li, Ning Wang, Zhen Li, Zhiyong Zhang, Rocky S. Tuan, Yangzi Jiang

**Affiliations:** ^1^ School of Biomedical Sciences The Chinese University of Hong Kong Shatin Hong Kong SAR China; ^2^ Institute For Tissue Engineering and Regenerative Medicine The Chinese University of Hong Kong Shatin Hong Kong SAR China; ^3^ Center For Neuromusculoskeletal Restorative Medicine Hong Kong Science and Technology Park Shatin Hong Kong SAR China; ^4^ AO Research Institute Davos Davos Switzerland; ^5^ Translational Research Centre of Regenerative Medicine and 3D Printing, Guangdong Province Engineering Research Center For Biomedical Engineering, State Key Laboratory of Respiratory Disease, The Third Affiliated Hospital Guangzhou Medical University Guangzhou Guangdong China; ^6^ Department of Orthopaedics & Traumatology The Chinese University of Hong Kong Shatin Hong Kong SAR China; ^7^ Key Laboratory for Regenerative Medicine Faculty of Medicine Ministry of Education The Chinese University of Hong Kong Shatin Hong Kong SAR China

**Keywords:** bioengineering, biomaterials, clinical trials, inflammation, MSCs

## Abstract

By virtue of their intrinsic immunomodulatory properties, mesenchymal stromal cells (MSCs) represent a promising therapeutic tool for immune‐related disorders. Research findings support that MSCs are involved in complex inflammatory pathologies by interacting with local immune cells. In addition to their immunomodulation ability, MSCs also contribute to cell‐mediated tissue regeneration due to their potential for multilineage differentiation. However, despite their accessibility, clinical translation of MSCs faces challenges, including their inherent heterogeneity, transient therapeutic effects, and microenvironment‐dependent functionality. This review provides an overview of current advances in MSC‐based therapies for immune‐related disorders, emphasizing Phase III and IV clinical trials and therapies approved by global regulatory agencies. Additionally, we highlight innovative engineering strategies designed to address the limitations of MSCs while enhancing their immunomodulatory capabilities. These approaches include: (1) cell pre‐treatment and genetic modification to improve therapeutic efficacy; (2) biomaterial‐mediated delivery systems for targeted sites; (3) MSC‐derived extracellular vesicle (EV)‐based therapeutics to amplify paracrine signaling; (4) induced pluripotent stem cell (iPSC)‐derived MSCs to overcome donor variability. By integrating these methodologies with ongoing clinical approaches, this review underscores the potential of engineered MSC immunomodulation in addressing inflammatory pathologies, bridging the gap between basic research and clinical application.

## Graphical abstract

Mesenchymal stromal cells (MSCs) show promise for treating immune‐related disorders through immunomodulation and tissue regeneration. This review gives a brief overview of current clinical approval of MSC therapies. It also discussed how bioengineering, including genetic modification, biomaterial delivery, extracellular vesicles, and iPSC‐derived MSCs, can enhance efficacy and bridge the gap between research and clinical application by overcoming the intrinsic limitations of MSCs.

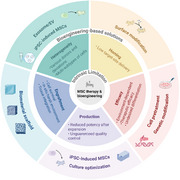

## 1 Introduction

Tissue regeneration is hindered by three major barriers: progenitor cell deficiency, chronic inflammation, and fibrosis [1]. Mesenchymal stromal/stem cells (MSCs), as multipotent cells, have been extensively investigated for their dual capacity of self‐renewal and multilineage differentiation, including but not limited to adipocytes, chondrocytes, osteoblasts, tenocytes, myoblasts, and fibroblasts. Their differentiation plasticity enables MSCs to replenish diverse somatic cell populations, a critical requirement for tissue repair and functional restoration [2]. In addition, MSCs have been shown to exhibit robust immunomodulatory activities, such as suppressing systemic inflammation in chronic inflammatory disorders and attenuating fibrosis progression [3], further endowing their candidacy in regenerative medicine applications.

**FIGURE 1:**
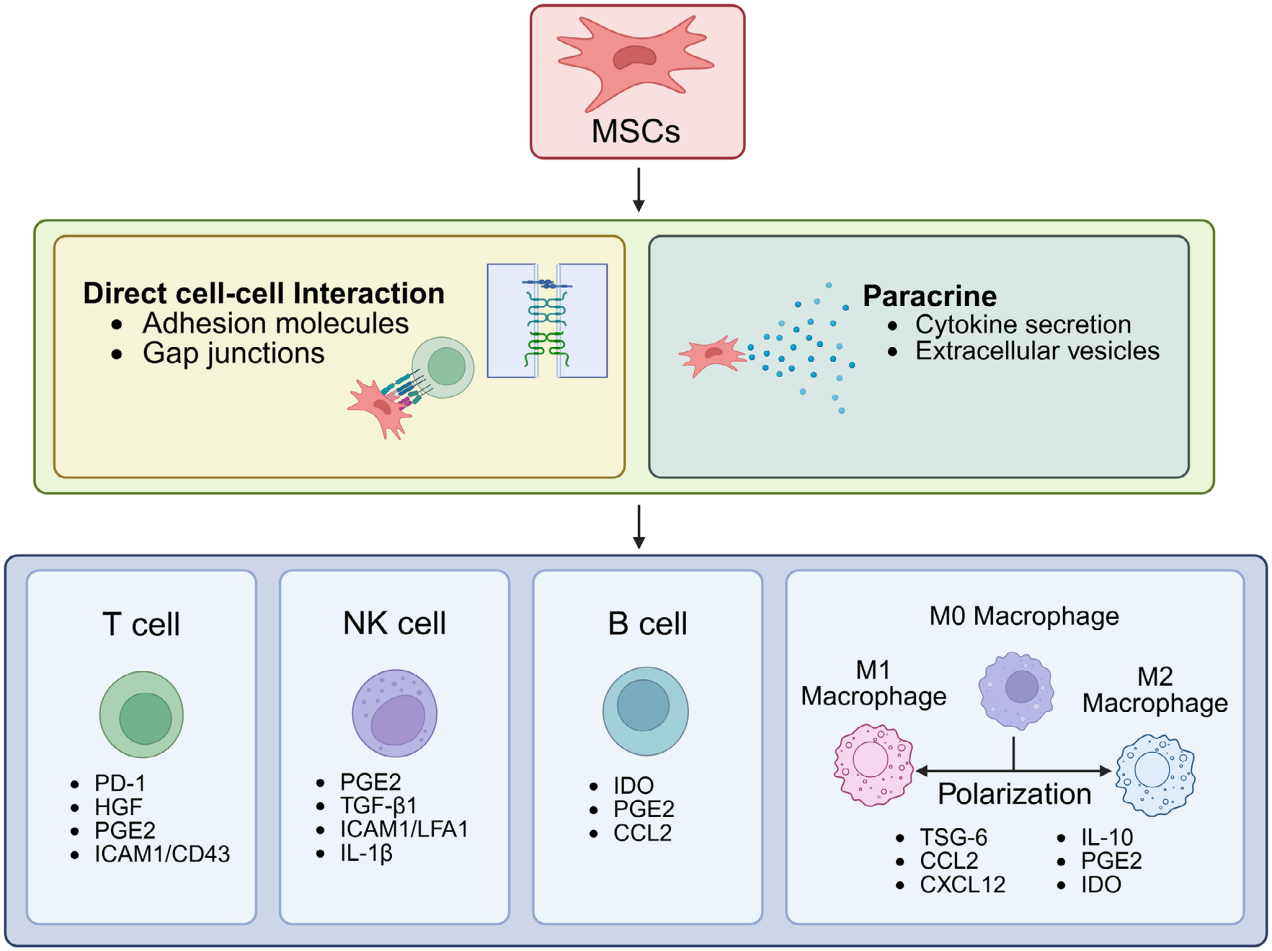
MSC interaction with immune cells. Schematic diagram of cell‐cell interactions between MSCs and immune cells. The interactions are mainly mediated by direct cell‐cell interaction or paracrine effects of MSCs.

The immunomodulatory potential of MSCs has been extensively explored over the past two decades, including clinical applications, such as cell‐based therapies for graft‐versus‐host disease (GVHD) [4]. This review highlights the immune‐suppressive properties of MSCs, which are mediated through intricate cross‐talk with the immune system via multiple key molecules (Figure 1). A critical mechanism underlying this immunosuppressive capacity is the low expression of human leukocyte antigen (HLA) class I and II molecules in MSCs, which minimizes MSC immunogenicity and reduces their recognition by the host immune system. MSCs exert their immunomodulatory effects through a network of molecules interacting with the adaptive immune system, including programmed death‐ligand 1 (PD‐L1) [5], indoleamine‐pyrrole 2,3‐dioxygenase (IDO) [6, 7], prostaglandin E2 (PGE2) [8], transforming growth factor‐β 1 (TGF‐β1) [9], and intercellular adhesion molecule‐1 (ICAM‐1) [10]. These molecules collectively suppress T and B cell activation while promoting regulatory T cell (Treg) differentiation. Concurrently, paracrine secretion of cytokines from MSCs, such as interleukin 4 (IL‐4) [11], interleukin‐1 receptor antagonist (IL‐1RA) [12], and IL‐10 [13], has proven to facilitate the polarization of macrophages toward the anti‐inflammatory M2 phenotype. Notably, the inflammatory microenvironment is recognized as a critical stimulator of MSC immunosuppressive activity [14, 15, 16], a process termed “licensing” which primes MSCs to enhance their functional responsiveness. This licensing mechanism underscores the dynamic nature of MSC immunomodulation and its dependence on local inflammatory cues [17, 18].

MSCs exhibit notable immunomodulatory activities through bidirectional crosstalk with both innate and adaptive immune systems, thereby enhancing their therapeutic utility in immune‐related pathologies such as autoimmune diseases and inflammatory disorders [19, 20, 21]. Their immune‐regulatory mechanisms are multi‐faceted, operating via cell‐cell contact and paracrine signaling (Figure 1). For instance, MSCs promote the polarization of macrophages toward the anti‐inflammatory M2 phenotype [22, 23], suppress the proliferation of activated T cells while inducing Treg cell differentiation [24, 25], and modulate natural killer (NK) cell cytotoxicity through secretion of type I interferons [26, 27]. Furthermore, the MSC secretome, in particular extracellular vesicles (EVs), plays a crucial role in immunomodulation by impairing antigen uptake in dendritic cells and stimulating anti‐inflammatory cytokine production [28, 29]. While MSCs demonstrate robust immunomodulatory potential, the corresponding capacities of other stem cell types, such as embryonic stem cells (ESCs), remain under‐explored and under‐utilized [30]. Owing to their unique immunosuppressive and anti‐inflammatory properties, MSCs have emerged as a promising therapeutic intervention tool in immune‐mediated diseases. Recent clinical applications of MSCs have demonstrated promising outcomes in treating conditions such as GVHD, systemic lupus erythematosus (SLE), and chronic inflammatory disorders, underscoring their translational value [31, 32, 33].

Chronic inflammation imposes a hostile microenvironment that impedes tissue repair by maintaining a prolonged pro‐inflammatory state, thereby delaying or impairing the healing process [1]. Beyond its local effects, chronic inflammation also drives other systemic immune‐related pathologies, including cardiovascular diseases, cancer, diabetes, chronic kidney disease, non‐alcoholic fatty liver disease, autoimmune disorders, and neurodegenerative diseases [34]. The robust immunomodulatory functions of MSCs, which alleviate chronic inflammation while fostering a regeneration‐conducive microenvironment, have promoted their candidacy in tissue engineering and regenerative medicine [35, 36]. In the realm of bone health, MSCs have demonstrated efficacy in mitigating chronic inflammatory bone loss by polarizing macrophages from a pro‐inflammatory to an anti‐inflammatory phenotype, thereby facilitating bone healing [37, 38]. Similarly, in preclinical models of Crohn's disease, MSCs attenuate chronic inflammation via dual mechanisms: reprogramming the local macrophages toward an anti‐inflammatory phenotype and suppressing T cell proliferation through PGE2 secretion. The combined action promotes durable tissue repair and functional recovery [39]. These multifaceted effects highlight the critical role of MSCs in maintaining immune homeostasis during tissue repair and regeneration.

**FIGURE 2:**
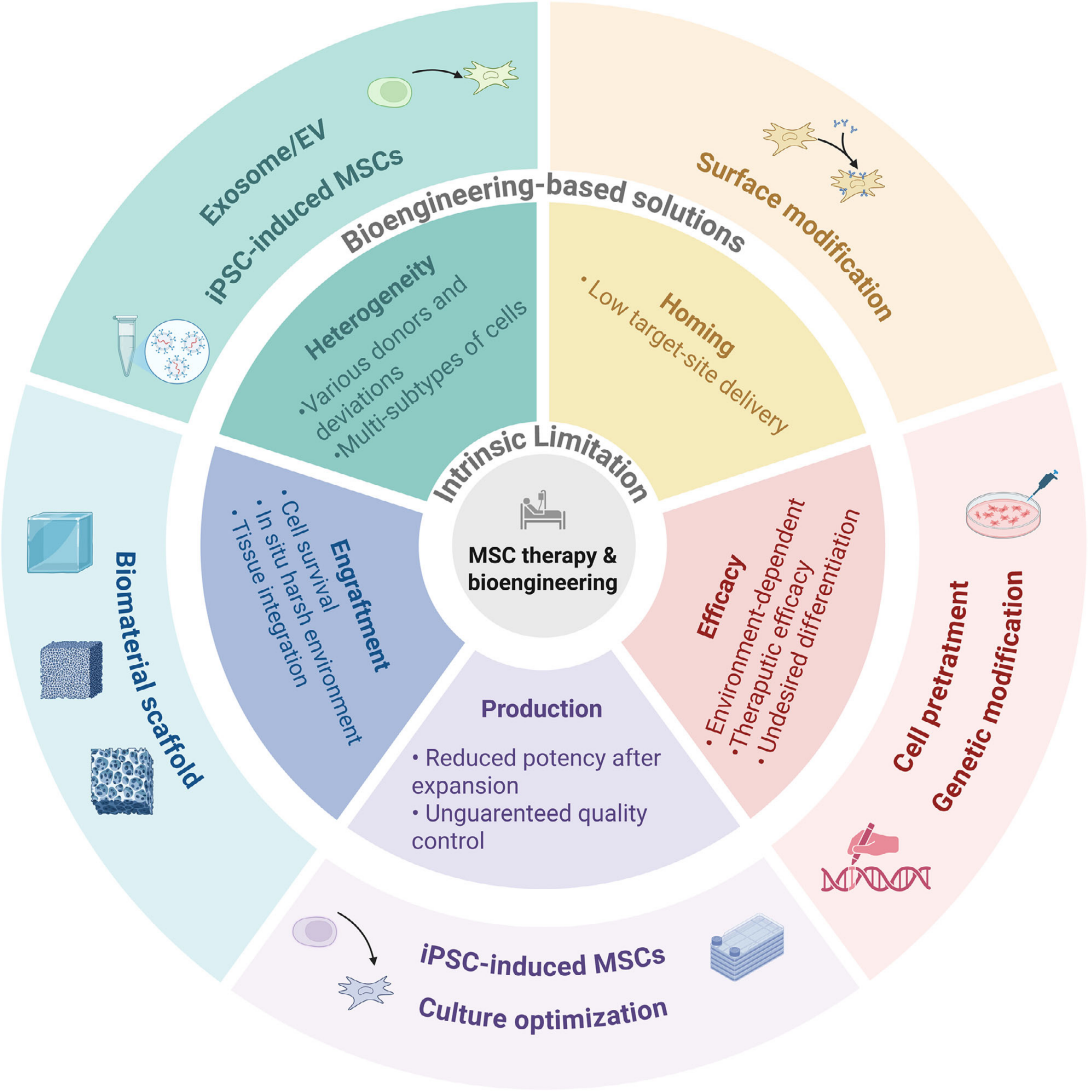
Limitations of MSC‐based therapies and potential solutions. Schematic illustration of key limitations hindering the clinical translation of MSC‐based therapies and corresponding bioengineering solutions.

Since the 1990s, scientists have been pursuing the utilization of MSC in clinical scenarios [40]. However, despite the strong immunomodulatory capabilities and regeneration potential of MSCs, their clinical application remains hindered by challenges such as poor engraftment, short‐term survival, transient efficacy, inconsistent therapeutic outcomes, and unreliable targeting [41]. Few therapies have shown reliable outcomes approved by regulatory agencies. Meanwhile, the challenges in administration route and side‐effects of MSCs‐based therapy such as transient fever, thromboembolism and local pain, along with minor problems including sleeplessness, constipation, and fatigue, prevented the further utilization of many potential applications [42, 43]. Based on the clinical problems, numerous engineering strategies are being developed to specifically address the limitations of MSC‐based therapies, with the objective of enhancing their clinical efficacy (Figure 2). Bioengineering approaches aim to address these limitations by improving MSC persistence, enhancing their homing ability, and boosting their immunomodulatory functions. By taking advantage of engineering approaches, specific immune‐related pathways and mechanisms can be regulated in MSCs. Genetic engineering techniques, including clustered regularly interspaced short palindromic repeats (CRISPR)‐Cas9 or viral transduction, are being employed to overexpress immunoregulatory molecules or silence pro‐inflammatory pathways [44]. Biomaterial‐based strategies utilize 3D scaffolds, hydrogels, or microcarriers to mimic the native MSC niche, and thus improve cell survival and paracrine signaling in vivo [45]. Preconditioning MSCs with cytokines or hypoxia enhances their secretory profile, boosting the production of anti‐inflammatory cytokines. Additionally, nanoparticle‐mediated delivery systems can be integrated to sustain localized release of MSC‐derived exosomes or therapeutics [46]. Surface engineering, such as conjugating homing ligands, improves MSC targeting to inflamed tissues [47]. These multidisciplinary approaches have been reported to synergistically enhance MSC interactions with immune cells, prolonged therapeutic effects, and address challenges such as poor engraftment and transient activity, thereby advancing their clinical application in autoimmune and inflammatory disorders. By taking advantage of the engineering approaches, we can manipulate MSCs into a more preferred style with reduced limitations, making MSC‐based therapy more feasible for clinical applications with higher consistency and efficacy.

This comprehensive review aims to advance the understanding and optimization of MSC‐based therapies and the immunomodulation of MSCs in tissue regeneration, particually in diseases caused by inflammation. This review first summarizes the current clinical applications of MSCs and recent developments in clinical trials, with a focus on immune‐related conditions. Subsequently, we discuss the diverse approaches and their corresponding mechanisms employed over the past five years to enhance the immunomodulatory capabilities of MSCs and their secreted EVs, which in turn facilitate tissue regeneration. These approaches encompass optimized cell culture protocols and preconditioning regimens, cell engineering techniques (e.g., genetic modification and cell reprogramming), and the use of biomaterial‐based scaffolds.

## 2 Clinical Trials and Applications

**FIGURE 3:**
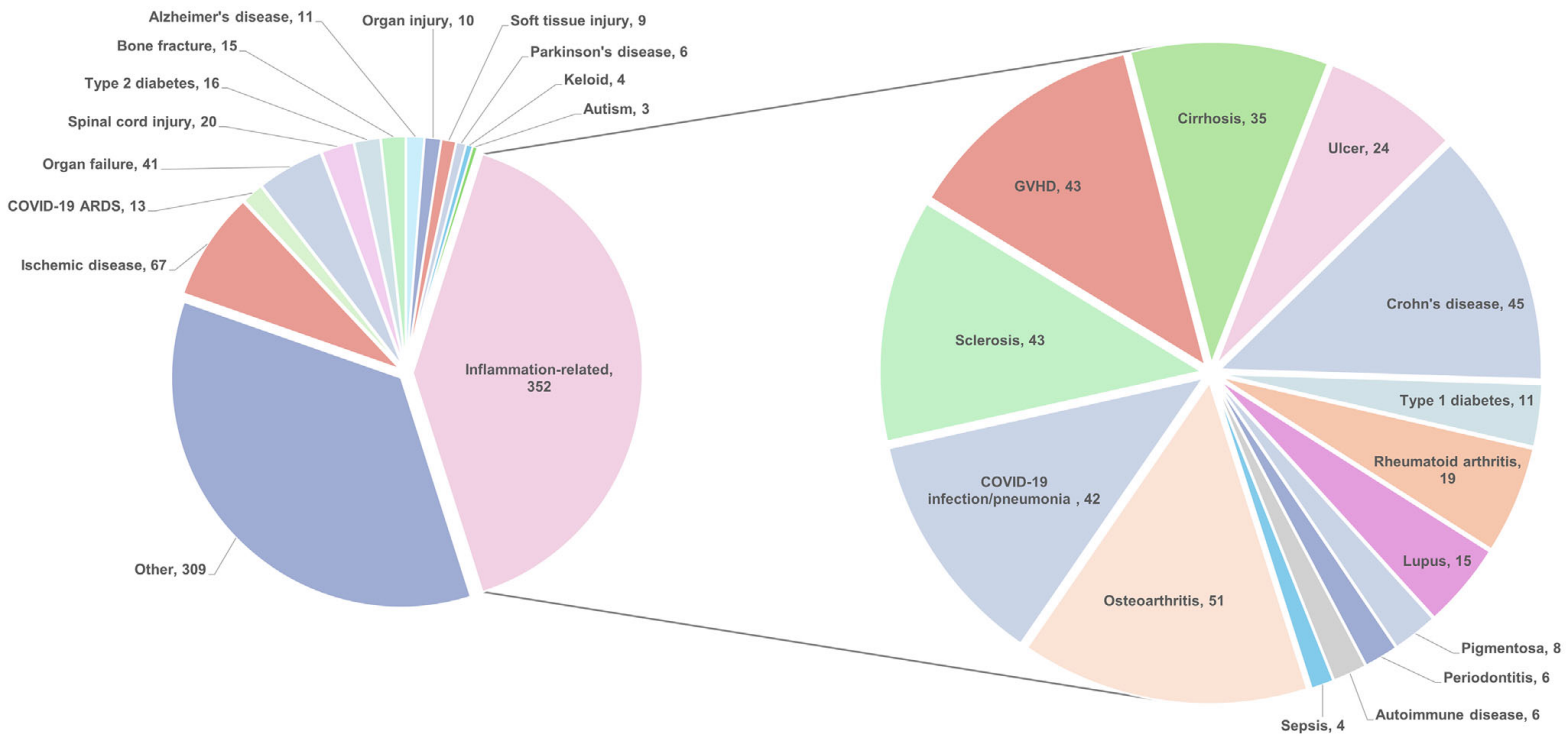
Statistics of current clinical trials of Phase 2 or higher. Targeted Condition of Phase 2 or higher MSC‐based clinical trials. As acquired from clinicaltrials.gov on Sep. 17, 2025, inputting “Mesenchymal stem cell” as the intervention keyword, 876 studies of phase 2 or higher clinical trials are identified. The right panel represents the detailed information of the “Inflammation‐related” cases in the left panel.

The application of MSCs has emerged as a focal point in regenerative medicine over the past two decades [4]. These cells are being investigated for their immunomodulatory properties across diverse clinical applications. As of Sep. 17, 2025, a search for “Mesenchymal stem cell” on ClinicalTrials.gov yielded 1614 studies, underscoring the sustained enthusiasm for their clinical exploration [48]. The leading geographical contributors to MSCs‐based clinical research include China, the United States, South Korea, Spain, and Iran, with over 60 countries currently participating in ongoing trials. Notably, immune and inflammation‐related diseases account for approximately 40% (340 trials) of the targeted conditions in 876 clinical trials reaching Phase II or beyond, emphasizing the therapeutic potential of MSCs in treating disorders such as GVHD, multiple sclerosis, Crohn's disease, osteoarthritis (OA), and rheumatoid arthritis (RA), as depicted in Figure 3. This section summarizes existing Phase III and Phase IV studies, comprising 47 trials. Completed trials are detailed in Table 1, while ongoing trials are listed in Table 2. Targeted conditions include, but are not limited to, OA, RA, Crohn's Disease, GVHD, and Amyotrophic Lateral Sclerosis (ALS).

**TABLE 1:** Completed phase 3 and phase 4 clinical trials targeting inflammation‐related disease and for regenerative purposes.

ID (Year/ Country)	Disease	Source of MSCs	Dosage	Patient number	Application methods	Primary outcome measurement	Results
NCT04243681 (2020/ India)	Decompensated Cirrhosis	(AT) HPCs (CD34+) combined with MSCs	NA	5	Injection in hepatic artery under fluoroscopic guidance	The safety of combination of hematopoietic and mesenchymal stem cell in patients of liver cirrhosis	Combined autologous HSC and MSCs infusion is safe in patients with decompensated liver cirrhosis but requires further evaluation in larger studies [49].
NCT03990805 (2022/ Korea)	OA	(AT) Adipose	100 million cells, once	260	Intra‐articular Injection	Change of WOMAC scores from baseline; Change of Visual Analog Scale (VAS) scores from baseline	Intra‐articular injection of autologous, culture‐expanded AD‐MSCs significantly improved pain and function in patients with K‐L grade 3 osteoarthritis [50].
NCT03818737 (2023/ United States)	OA	(AT/AL) Bone Marrow/Umbilical Cord/Adipose	20 million cells in 4 mL for UC‐MSCs	480	Intra‐articular Injection	Change in VAS‐pain Score and KOOS‐Pain Subscale Score	No serious adverse events were reported. At one‐year post‐injection, the orthobiologic was not superior to CSI for knee osteoarthritis [51].
NCT01873625 (2013/ Iran)	Rheumatoid Arthritis	(AT) Bone Marrow	40 million, once	60	Intra‐articular implantation	Pain; physical activity; walking487706 distance	Intra‐articular MSC implantation offered significantly greater pain relief than placebo over six months [52].
NCT01803347 (2018/ Spain)	Perianal Fistula	(AT) Adipose	100 million, and another dose on the 16th week if needed	80	Local injection	Safety/efficacy	Autologous ASCs for the treatment of cryptoglandular perianal fistula is safe and can favor long‐term and sustained fistula healing [53].
NCT01541579 (2019/ Austria)	Perianal Fistulizing Crohn's Disease	(AL) Adipose	120 million cells	278	intralesional injection	Combine remission of perianal fistulising Crohn's	MSCs was safe and effective in closing external openings, compared with placebo, after one year [54].
NCT01233960 (2021/ United States)	Crohn's Disease	(AL) Bone Marrow	200 million cells	73	Intravenous injection	Crohn's Disease Activity Index (CDAI) at or below 150 and increase in IBDQ	No results posted
NCT03370874 (2023/ Korea)	Diabetic Foot Ulcer	(AL) Adipose	Hydrogel sheet containing allogenic adipose‐derived mesenchymal stem cells	150	NA	Proportions of subjects who achieved complete wound closure	No results posted
NCT00543374 (2021/ United States)	Crohn's Disease	(AL) Bone Marrow	600 million cellsor 1200 million cells in total, twice per week, 2 weeks	98	Intravenous injection	Duration of clinical benefit (Crohn's disease activity index); Re‐induction of clinical benefit (Crohn's disease activity index)	No results posted
NCT00482092 (2022/ United States)	Crohn's Disease	(AL) Bone Marrow	600 million cells or 1200 million cells, daily, four days	330	Intravenous injection	Disease remission (CDAI at or below 150)	No results posted
NCT05939817 (2023/ Indonesia)	Keloid	(AL) Umbilical Cord	2 million cells/mL/cm^3^ according to keloid size	24	Local injection	Type 1:3 collagen ratio reduction; IL‐10 levels increase	No results posted
NCT05887804 (2023/ Indonesia)	Keloid	(AL) Umbilical Cord	2 million cells/mL/cm^3^ according to keloid size	24	Local injection	Type 1:3 collagen ratio reduction; IL‐10 levels increase	Both UC‐MSCs and UC‐CM are more effective than TA for keloid therapy, showing greater reduction in keloid volume, symptom severity, and the type 1:3 collagen ratio, as well as a greater increase in IL‐10 levels [55].
NCT04738981 (2023/ China)	Acute GVHD	(AL) Umbilical Cord	1 million cells/kg, once a week, 4 weeks	130	Intravenous injection	Rate of complete remission	For steroid‐refractory aGVHD after allo‐HSCT — particularly following HID HSCT — adding MSCs to basiliximab improved 4‐week complete response rates and survival compared to basiliximab alone, without increasing toxicity [56].
NCT00562497 (2022/ United States)	Acute GVHD	(AL) Bone Marrow	2 million cells/kg, twice per week in first 2 weeks, once per week for next 2 weeks	192	Intravenous injection	Percentage of Participants with Treatment Success	Failed
NCT00366145 (2022/ United States)	Acute GVHD	(AL) Bone Marrow	2 infusions of 2 million cells/kg per week, weeks	260	Intravenous injection	Percentage of Participants achieving Complete Response of Greater Than or Equal to 28 Days Duration	Failed [57]
NCT06469411 (2024/ Iran)	GVHD	(AL) Placenta	6 doses of secretome of MSCs (400 µg protein/mL) per week, 6 weeks	60	Intravenous injection	Liver involvement; Intestine condition; Skin rash	No results posted
NCT04877067 (2021/ Turkey)	Toxic Optic Neuropathy	(AL) Wharton's Jelly	2‐6 × 10^6^ cells in 1.5 mL	18	Eye subtenon space injection	ETDRS visual acuity	The injection demonstrated retinal regeneration and remained safe after 6 months, with no serious side effects reported [58].
NCT05800301 (2021/ Turkey)	Toxic Optic Neuropathy	(AL) Wharton's Jelly	2‐6 × 10^6^ cells in 1.6 mL	80	Eye subtenon space injection	Fundus autofluorescence surface area	The treatment significantly slows disease progression over three years compared to its natural course [59].
NCT03766217 (2020/ Brazil)	Alveolar Cleft	(AT) Dental Pulp	1 million cells	62	Biomaterial filling	Alveolar bone filling rate	The therapy demonstrated effective bone healing with good safety and feasibility over 6 to 12 months [60].
NCT03418233 (2021/ Poland)	Chronic Ischaemic Heart Failure	(AL) Wharton's Jelly	3 × 10^7^ cells	105	Transcoronary injection	Left ventricle ejection fraction	No results posted
NCT03404063 (2021/ Poland)	Acute Myocardial Infarction	(AL) Wharton's Jelly	3 × 10^7^ cells	105	Transcoronary injection	Reduction of infarct size	No results posted
NCT03325504 (2024/ Spain)	Long Bone Non‐union	(AT) Bone Marrow	1–2 × 10^7^ million cells	46	Biomaterial filling	Bone consolidation	Result yet posted [61]
NCT03280056 (2024/United States)	Amyotrophic Lateral Sclerosis	(AT) Bone Marrow	1–1.25 × 10^8^ cells, once every 8 weeks, 3 times	196	Intrathecal administrations	The ALS Functional Rating Scale–Revised	Treatment for 28 weeks led to significant advancements in cerebrospinal biomarkers linked to neuroinflammation, neurodegeneration, and neurotrophic support [62].

Abbreviations: AL, Allogenic; AT, Autologous; CDAI, Crohn's Disease Activity Index; CSI, corticosteroid injection; GVHD, Graft‐versus‐host disease; HSCT, Hematopoietic stem‐cell transplantation; KOOS, Knee Injury and Osteoarthritis Outcome Score; SR‐aGVHD, Steroid‐refractory acute graft‐versus‐host disease; NA, Not applicable; MSCs, Mesenchymal Stem Cell; VAS, Visual Analogue Scale.

The data are acquired from clinicaltrials.gov.

**TABLE 2:** To be completed phase 3 and phase 4 clinical trial targeting inflammation‐related disease.

ID (Country)	Disease	Source of MSCs	Status	Dosage	Planned recruited case	Application methods	Primary outcome measure
NCT03631589 (China)	Acute GVHD	NA	NA	NA	50	NA	Complete and partial response rate
NCT03389919 (Russia)	Acute GVHD	(AL) Bone Marrow	NA	NA	20	Intraosseous implantation	Engraftment
NCT02241018 (China)	Acute GVHD	(AL) Bone Marrow	NA	2 million cells/kg, once per week, 4 weeks; if reach partial response, give another cycle.	200	NA	The efficacy of treatment for steroid‐resistant aGVHD
NCT04629833 (France)	Acute GVHD	(AL) Bone Marrow	Recruiting	Participants will receive weekly infusions of MC0518 (1‐2 million cells/kg) for 4 weeks. Those with a partial response on Day 28 will receive two additional infusions on Days 29 and 36.	210	Intravenous injection	Overall Response; Overall Survival
NCT02291770 (China)	Chronic GVHD	NA	NA	2 million cells/kg, twice per week in first 2 weeks, once per week for next 2 weeks	130	Intravenous injection	Proportion of patients responding to treatment of cGVHD with MSCs
NCT01526850 (China)	Chronic GVHD	NA	NA	10–20 million, once per week, 4 weeks, continue if needed	100	Bone marrow injection	The total Response rate defined as patients with complete and partial response
NCT06149832 (China)	Oral Chronic GVHD	(AL) Umbilical Cord	Recruiting	MSCs 1 million/ml 4 times/day, 2 weeks	38	Dressing in mouth	Oral cGVHD improves condition
NCT06731192 (China)	Alport syndrome	(AL) Umbilical cord	Not yet recruiting	Two injections, 14 days between injections, 20 million cells/Kg	40	Intravenous injection	Urine protein remission rate
NCT02809781 (China)	Ankylosing Spondylitis	Bone Marrow	NA	1 million cells/kg, once per week in first 4 weeks, once per 2 weeks for next 8 weeks	250	Intravenous drop	The Assessment of Spondyloarthritis International Society (ASAS)20 response
NCT03112122 (Italy)	Bone Marrow Edema	(AT) Bone Marrow	Terminated (difficulty in recruiting)	NA	NA	Subchondral anterograde drilling	VAS
NCT04018729 (Brazil)	Chronic obstructive pulmonary disease	Bone Marrow	Recruiting	NA	34	NA	All‐cause death; Number of participants with worsening of dyspnea; Number of participants with respiratory functional worsening; Impairment of exercise capacity; Increased oxygen use
NCT04612465 (Korea)	Crohn's Disease	(AT) Adipose	Recruiting	10 million cells of ASC per 1 cm^2^ of the surface area of the fistula	36	In location injection	Proportion of subjects who are completely blocked fistula
NCT04569409 (Korea)	Foot Ulcer	(AL) Adipose	Active, not recruiting	Hydrogel sheet containing allogenic adipose‐derived mesenchymal stromal cells	104	NA	Proportions of subjects who achieved complete wound closure
NCT04247945 (China)	GVHD	(AL) NA	Recruiting	NA	120	NA	Survival Rate
NCT05216562 (Indonesia)	Hyper‐inflammation in COVID‐19	EXOSOME‐MSCs (resource not mentioned)	NA	NA	60	Intravenous injection	Time to clinical improvement (days)
NCT06893250 (Norway)	Knee osteoarthritis	(AT) Adipose	Enrolling by invitation	NA	160	Intra‐articular injection	KOOS, VAS, WOMAC score
NCT06716281 (China)	Knee osteoarthritis	(AL) Umbilical cord	Recruiting	50 million cells with a volume of 2.0 mL	398	Intra‐articular injection	WOMAC score; cartilage damage area
NCT05080465 (Ukraine)	Liver Cirrhosis	(AT) Bone Marrow	Active, not recruiting	A single dose of 0.5 to 1 million cells/kg	700	Peripheral venous injection	MR Elastography; The level of serum alanine aminotransferase (ALT); Clinical Examination; The level of glomerular filtration rate (GFR)
NCT01854125 (China)	Liver Cirrhosis	(AL) Bone Marrow	NA	1 million cells/kg	30	Infused via liver artery	change in immune function, liver function in blood
NCT05631717 (China)	Lupus Nephritis	(AL) Umbilical Cord	Recruiting	1 million cells/kg body weight, once	40	Intravenous injection	Response rates in both groups (Complete response and Partial response)
NCT06570291 (China)	OA	(AL) Adipose	Recruiting	NA	520	Intra‐articular injection	WOMAC score, MRI quantitative analysis of articular cartilage
NCT05086939 (Spain)	OA	(AT/AL) Bone Marrow	Active, not recruiting	40 million/4 mL	124	Intra‐articular injection	Range of motion; Pain self‐assessment; Knee Osteoarthritis; Functional response; X‐ray changes of osteoarthritis; Radiological response using nuclear magnetic resonance imaging [63]
NCT04427930 (Korea)	OA	(AT) Adipose	Active	NA	129	Intra‐articular injection	Adverse Events
NCT04351932 (Ecuador)	OA	(AT) Adipose/Bone Marrow	NA	10 cc	54	Intraarticular injection	Knee pain assessed by Visual Analogue Scale; WOMAC score [64]
NCT04230902 (Lebanon)	OA	(AT) Adipose	NA	NA	48	Intraarticular injection	Comparing the total pain score pre‐ and post‐injection differences between the two arms; Comparing MOAKS pre‐and post‐injection differences between the two arms [65]
NCT05660824 (Switzerland)	OA	(AT) Stromal Vascular Fraction (SVF) containing MSCs	Not yet recruiting	1.5 mL SVF	119	Intraarticular injection	The absolute difference between the treatment and control groups on the Single Assessment Numeric Evaluation
NCT05783154 (Bangladesh)	OA	(AT) Adipose	NA	NA	84	Intraarticular injection	Changes in the Articular Cartilage Defect; Changes in Femoral Cartilage thickness; Changes in the Pain Intensity; Changes in Physical functioning
NCT04368806 (United States)	OA	(AT) Adipose	NA	NA	140	Intraarticular injection	WOMAC; VAS
NCT02138331 (Egypt)	Type 1 Diabetes	(AL) Umbilical Cord MSCs Macrovesicles	NA	First dose: purified exosomes (40‐180 nm) from a supernatant of 1.22‐1.51 million cells/kg; second dose: microvesicles (180‐1000 nm) from an equivalent supernatant dose.	20	Intravenous injection	Total daily insulin dose
NCT01157403 (China)	Type 1 Diabetes	(AT) Bone Marrow	NA	2.5 million cells/kg	80	Intravenously injection	C peptide release test

Abbreviations: AL, Allogenic; ALT, Alanine aminotransferase; ASAS, Assessment of Spondyloarthritis International Society; AT, Autologous; GFR, Glomerular filtration rate; GVHD, Graft‐versus‐host disease; KOOS, Knee Injury and Osteoarthritis Outcome Score; MOAKS, MRI Osteoarthritis Knee Score; MRI, Magnetic resonance imaging; MSCs, Mesenchymal Stem Cell; NA, Not applicable; SVF, Stromal vascular fraction; WOMAC, Western Ontario and McMaster Universities Osteoarthritis Index; VAS, Visual Analogue Scale.

The data are acquired from *clinicaltrials.gov*.

Here are some details and discussion of Phase III trials with reported outcomes and approved drugs.

### 2.1 Phase III Outcomes for OA Treatment

OA, a degenerative joint disorder characterized by cartilage degradation, bone remodeling, and chronic inflammation, lacks curative treatments, making MSC‐based therapy a promising strategy due to its potential to repair damaged tissue, reduce inflammation, and potentially restore joint function. A multicenter Phase III clinical trial conducted in Korea evaluated the safety and efficacy of autologous adipose‐derived mesenchymal stem cells (AD‐MSCs) in patients with knee OA [50]. The clinical trial enrolled a total of 261 patients with K‐L Grade 3 knee OA, randomized in a 1:1 ratio into the AD‐MSC treatment group (n = 131) and placebo control group (*n* = 130). At six‐month post‐single intra‐articular injection of 100 million autologous AD‐MSCs, primary efficacy outcomes assessing pain and functional status demonstrated statistically significant improvements in the AD‐MSC treatment group compared to controls. Pain level measured by the 100‐mm visual analogue scale (VAS) improved substantially in the AD‐MSC group (mean change of 25.2 points vs. 15.5 points in the control group). Similarly, functional improvements measured by the Western Ontario and McMaster Universities Osteoarthritis Index (WOMAC) showed greater change in the AD‐MSC treatment group (21.7 points improvement) compared to the control group (14.3 points improvement). However, magnetic resonance imaging (MRI) analysis failed to demonstrate significant structural improvement. These findings establish autologous AD‐MSC intra‐articular injections as a promising therapeutic option for K‐L Grade 3 knee OA, although its structural disease‐modifying potential remains inconclusive and requires further investigation.

A Phase III clinical trial in the United States in 2023 evaluated the efficacy and safety of three cell‐based therapies for knee OA compared to standard corticosteroid injection (CSI) in 480 patients [51]. The trial evaluated the efficacy and safety of three different cellular injections—autologous bone marrow aspirate concentrate (BMAC), autologous stromal vascular fraction (SVF), and allogenic human umbilical cord tissue MSCs (20 million cells in 4 mL plasmalyte A + 5% human serum albumin). The primary endpoints measured change in pain scores (assessed via the VAS) and functional outcomes (via the Knee Injury and Osteoarthritis Outcome Score, KOOS) at 12 months post‐treatment. While the trajectory of VAS score reductions differed slightly among the cellular therapy groups over time, there were no statistically significant differences in mean VAS improvement between the three cell therapies and the CSI group at the 12‐month follow‐up. Similarly, KOOS pain score analysis showed comparable improvements across all groups, failing to demonstrate statistically superior efficacy for any cellular treatment over CSI. The findings indicate that none of the tested cellular therapies (BMAC, SVF, or allogenic MSCs) provided a clinically meaningful advantage over the standard corticosteroid injections for knee OA over a one‐year period [51].

Cartistem is a drug approved by the Korean Ministry of Food and Drug Safety (MFDS) in 2012 for the treatment of severe OA, demonstrating optimistic efficacy compared to prior mentioned trials [66]. The treatment involves intra‐articular injection of human umbilical cord blood‐derived cells at a dose of 2.5 × 10^6^ cells /50 µL/cm^2^, adjusted according to the size of the defect. A 7‐year longitudinal follow‐up study has confirmed the sustained efficacy and durability of Cartistem post‐treatment [67]. Furthermore, the developer is investigating its co‐administration with hyaluronan following market approval. Preliminary results from a 5‐year follow‐up study highlight the therapeutic potential of this combination in addressing cartilage defects, suggesting synergistic benefits [68].

### 2.2 Phase III Outcomes for GVHD Treatment

GVHD is a life‐threatening complication after allogenic tissue/organ transplantation caused by donor immune cells attacking the recipient's tissues, obviating the need for MSC‐based therapies to modulate immune dysregulation and mitigate organ damage. GVHD is a well‐targeted indication for MSC therapies owing to its high lethality and limited secondary treatment options [69]. A pivotal Phase III trial in China centered on MSC‐based intervention for steroid‐resistant acute graft‐versus‐host disease (SR aGVHD), enrolling 198 patients randomly assigned to receive either bone marrow‐derived MSCs (BM‐MSCs) combined with basiliximab and a calcineurin inhibitor, or a standard therapy without MSCs. The MSCs group received intravenous infusions of 1 × 10^6^ cells/kg once per week. By day 28, the MSC cohort achieved an overall response (OR) rate of 82.8% (durable OR: 78.8%), significantly surpassing the control group's 70.7% OR and 64.6% durable OR, respectively, thereby affirming the efficacy of MSCs in this refractory clinical scenario [70].

Notably, the Chinese drug PLEB‐001 (trade name: Amimestrocel; NMPA approval number CXSS2400062), developed by Platinum Life Excellence Biotech, received formal approval from the China National Medical Products Administration (NMPA) in January 2025 [71]. This drug utilizes human umbilical cord‐derived MSCs as a treatment for acute GVHD in patients who have failed to respond to hormone therapy, underscoring the rapid advancement of stem cell therapies in the Asia‐Pacific region [72, 73, 74]. The drug's regulatory pathway represents a notable acceleration in the application of stem cells, namely it bypassed the traditional Phase I clinical trial stage following submission of its Stem Cell Clinical Research Report to the Center for Drug Evaluation (CDE), proceeding directly to Phase II trials [71]. Between 2020 and 2022, the Phase II clinical trial enrolled 96 patients to assess both efficacy and safety parameters [75, 76, 77]. Building on this, a Phase III trial was initiated in 2023 with 60 enrolled participants [78]. However, critical trial outcomes for both stages remain confidential pending further disclosure by the sponsor.

Remestemcel‐L is a mesenchymal precursor cell (MPC) product derived from allogenic human bone marrow‐derived MSCs [79], commercially known as Prochymal or Ryoncil. This therapy has garnered global regulatory recognition, having secured approval from the U.S. Food and Drug Administration (FDA) and Health Canada for the treatment of steroid‐refractory aGVHD following robust evidence from Phase III clinical trials [33, 80, 81, 82]. Remestemcel‐L shows its potential in multiple diseases, and the drug's regulatory trajectory demonstrates its clinical significance. In 2010, it became the first MPC‐based therapy licensed for manufacturing and supply in Australia by the Therapeutic Goods Administration (TGA) [83]. Concurrently, the U.S. FDA granted Orphan Drug Designation to Prochymal for type I diabetes mellitus [84]. By 2012, Health Canada issued a conditional approval for GVHD treatment (need more data) [85], and this was followed by approvals from New Zealand's Medsafe and Japan's Pharmaceuticals and Medical Devices Agency (PMDA, named as Temcell) in 2016, with clinical outcomes comparable to international trials [86, 87]. Most recently, in late 2024, Remestemcel‐L‐rknd (Ryoncil) achieved landmark FDA approval as the first MSC‐based drug for steroid‐refractory aGVHD [88]. Beyond GVHD, Remestemcel‐L exhibits therapeutic potential in other diseases and conditions [89]. Ongoing clinical trials (ClinicalTrials.gov ID: NCT00482092, NCT00366145, NCT01233960, NCT00562497, NCT00543374) have investigated its efficacy in Crohn's Disease and aGVHD [90, 91, 92, 93], with preliminary evidence suggesting immunomodulatory benefits in inflammatory disorders [94].

### 2.3 Approved MSC Therapies

Notwithstanding the numerous registered MSC trials, the number of approved MSC‐based therapies remains limited. As of 2023, 12 drugs have shown promising potential and have received approvals from regulatory agencies in various countries or regions [80, 85]. Among these, six therapies specifically target immune and inflammatory diseases, including Cupistem, Cartistem, Remestemcel‐L, Temcell HS, Alofisel, and Mesestro‐Cell (see details in Table 3).

**TABLE 3:** Approved MSC‐based drug (2023 data).^a^

Name/Year	Company	Origin	Use	Description	Dosage	AT/AL	Charge
Cupistem/2012	Anterogen (Republic of Korea)	Republic of Korea MFDS	Crohn's fistula	Human‐adipose‐tissue‐derived MSCs	Fistula diameter: (a) ≤1 cm (3 × 10^7^ MSCs in 1 mL) (b) 1 < X< 2 cm (6 × 10^7^ MSCs in 2 mL)	AT	USD 5000 per treatment
Cartistem/2012	Medipost (Republic of Korea)	Republic of Korea MFDS	Knee OA (ICRS grade IV)	Human‐umbilical‐cord‐blood‐derived MSCs	7.5 × 10^6^ cells/vial (depending on the size of the lesion)	AL	USD 21 000 per treatment
Reme‐stemcel‐L/2015	Mesoblast, Ltd. (Australia)	US FDA	Acute and refractory GvHD for pediatric patients	Human‐bone‐marrow‐derived MSCs	IV administration: Low (2 million cells/kg) High (8 million cells/kg)	AL	USD 200 000 per treatment
Temcell HS/2015	JCR Pharmaceuticals (Japan)	Japan PMDA	Acute and refractory GvHD	Human‐bone‐marrow‐derived MSCs	IV infusion of 2 million cells/kg (each bag contains 72 million cells in 18 mL of saline); 4 mL per minute twice weekly in intervals of 3 days or more for 4 weeks	AL	USD 7600 per bag
Alofisel/2018	TiGenix (US) and Takeda (UK)	EMA	Complex perianal fistulas in CD	Human‐adipose‐tissue‐derived MSCs	Vial: 30 million MSCs/6 mL Treatment: 4 vials	AL	USD 47 485 per treatment
Mesestro‐Cell/2018	Cell Tech Pharmed (Iran)	Iran FDA	OA	Bone‐marrow‐derived MSCs	A minimum intra‐articular injection of 2 × 10^7^ cells/knee; in total, 4 × 10^7^ cells for both knees	AT	NA

Abbreviations: AL, allogeneic; AT, autologous; CD, Crohn's disease; EMA, European Medicine Agency; FDA, Food and Drug Administration; GvHD, graft versus host disease; ICRS, International Cartilage Repair Society; IV, intravenous; MSCs, mesenchymal stem cells; NA, not available; OA, osteoarthritis; PMDA, Pharmaceuticals and Medical Devices Agency; UK, United Kingdom; US, United States.

^a^ Table is reproduced with permission [80]. Copyright 2023, MDPI.

Extensive clinical trials highlight the positive outcomes and promising future of MSC‐based therapies. However, certain studies reveal the inefficacy of MSCs in specific diseases and clinical scenarios. With only 1% to 10% of drugs progressing from Phase I trials to market approval [48, 95], this underscores the likelihood of limited success in upcoming approvals. Notably, the recent MSC‐based therapy approvals by the China NMPA and the US FDA suggest a potential acceleration of regulatory progress. Efforts must focus on enhancing MSC characterization, mitigating limitations, and addressing barriers to clinical application. Meanwhile, the clinical use of MSC‐derived EVs is advancing, with 42 trials registered on ClinicalTrials.gov investigating MSC‐EV for diverse diseases [48]. This expands the therapeutic applications of MSCs beyond direct cell therapy.

### 2.4 Limitations of MSCs Therapy in Clinical Application

Although the clinical translation of MSC‐based therapies has advanced considerably, only a limited number have successfully completed all phases of clinical trials (Tables 1 and 2) and received formal regulatory approval (Table 3). The path toward widespread clinical adoption of native MSCs, engineered MSCs, and their EV derivatives remain fraught with multifaceted challenges, including the long and complicated bench‐to‐bedside translation procedures.

The major concerns that halting the clinical translation of MSC includes inconsistent therapeutic efficacy, poor post‐transplantation cell survival, donor‐ and process‐driven heterogeneity, environment‐dependent efficacy, undesired in situ differentiation, challenges in establishing scalable, Good Manufacturing Practice (GMP)‐compliant production systems, and an incompletely defined mechanism of action [96, 97]. Variability in tissue sources, differences in isolation techniques, and non‐uniform culture conditions contribute to substantial batch‐to‐batch heterogeneity, raising concerns about the reproducibility and reliability of clinical outcomes. Moreover, the mechanistic basis of MSC‐mediated therapeutic effects remains incompletely elucidated, as MSC functionality is highly context‐dependent, shaped by dynamic interactions with the host microenvironment—including inflammatory signals, hypoxia, and crosstalk with resident immune and stromal cells—which differ markedly across patients and disease states. This microenvironmental plasticity underlies the variable efficacy observed in clinical trials and underscores the urgent need for a deeper understanding of MSC–host interactions to inform rational bioengineering approaches—such as microenvironment‐responsive design, precision delivery systems, or synergistic combination therapies—that can maximize therapeutic predictability and clinical impact. Meanwhile, standardized protocols for the isolation, expansion, characterization, and quality control of MSCs and EVs, while maintaining their efficacy and bioactivity, are not yet well‐established [98].

Bioengineering offers a powerful and integrative strategy to overcome the above limitations (Figure 2). For instance, genetic modifications [99, 100] and preconditioning protocols [44, 101] can enhance MSC survival, immunomodulatory capacity, and responsiveness to disease‐specific microenvironments. Additionally, surface engineering [102, 103] endows MSCs with enhanced targeting ability, which eventually improves the efficacy and clinical outcomes of the therapies. Biomaterial‐assisted delivery systems create a more preferable environment for MSCs, improving targeted retention and protecting cells from the hostile conditions, such as inflammatory microenvironment [104]. Engineered EVs provide cell‐free alternatives with enhanced safety, stability, and storage feasibility. Current bioengineer methods under investigation to enhance the in vitro production of MSCs and MSC‐EVs often involve cell priming [105], modified culture environment [106, 107], or 3D‐cell culture [108, 109]. Additionally, induced MSCs (including induced pluripotent stem cell (iPSC)‐derived MSCs), as an emerging approach, provide a consistent cell resource of MSCs [110]. By taking advantage of designed and standardized induction protocols, the approach can help tackle problems of inconsistent large‐scale production and heterogeneity of MSCs. Collectively, these innovations aim to transform MSCs from empirically administered cellular products into precisely designed, reproducible, and controllable therapeutic agents, thereby narrowing the gap between preclinical promises and clinically viable, regulatorily compliant regenerative therapies.

While overcoming the clinical limitations of MSCs, it is essential to select the administration methods of MSCs based on the specific disease conditions. Intravenous (IV) administration is commonly used, leading to initial cell accumulation in the lungs, followed by redistribution to the liver, spleen, and kidneys. This biodistribution pattern is crucial for systemic effects but may limit targeted delivery. For localized treatments, intramuscular, intraarticular, and intradermal routes are employed [111]. In the context of ocular injuries, subconjunctival and IV administration have demonstrated superior efficacy in reducing inflammation and promoting tissue repair compared to topical or intraperitoneal methods [112]. The choice of administration route is also influenced by the source of MSCs. Injected BM‐MSCs show greater efficacy in liver disease when administered via the hepatic artery versus peripheral vein administration [113]. Furthermore, the administration route can affect the therapeutic outcomes of MSC‐derived EVs, with topical application proving most effective in skin conditions owing to its ability to localize in the stratum corneum and reinforce the skin barrier [114]. When it comes to respiratory‐related disease, administration of MSCs and MSC‐derived EVs via inhalation often shows better performance than IV injection in terms of targeting and efficacy [115, 116], which EVs are often the better choice of inhalation administration due to better stability [116]. Thus, the administration method must be selected based on the specific clinical context and desired outcome in the application of MSC‐based therapy.

## 3 Current Research Approach of MSCs Engineering to Modify Immunomodulation

**FIGURE 4:**
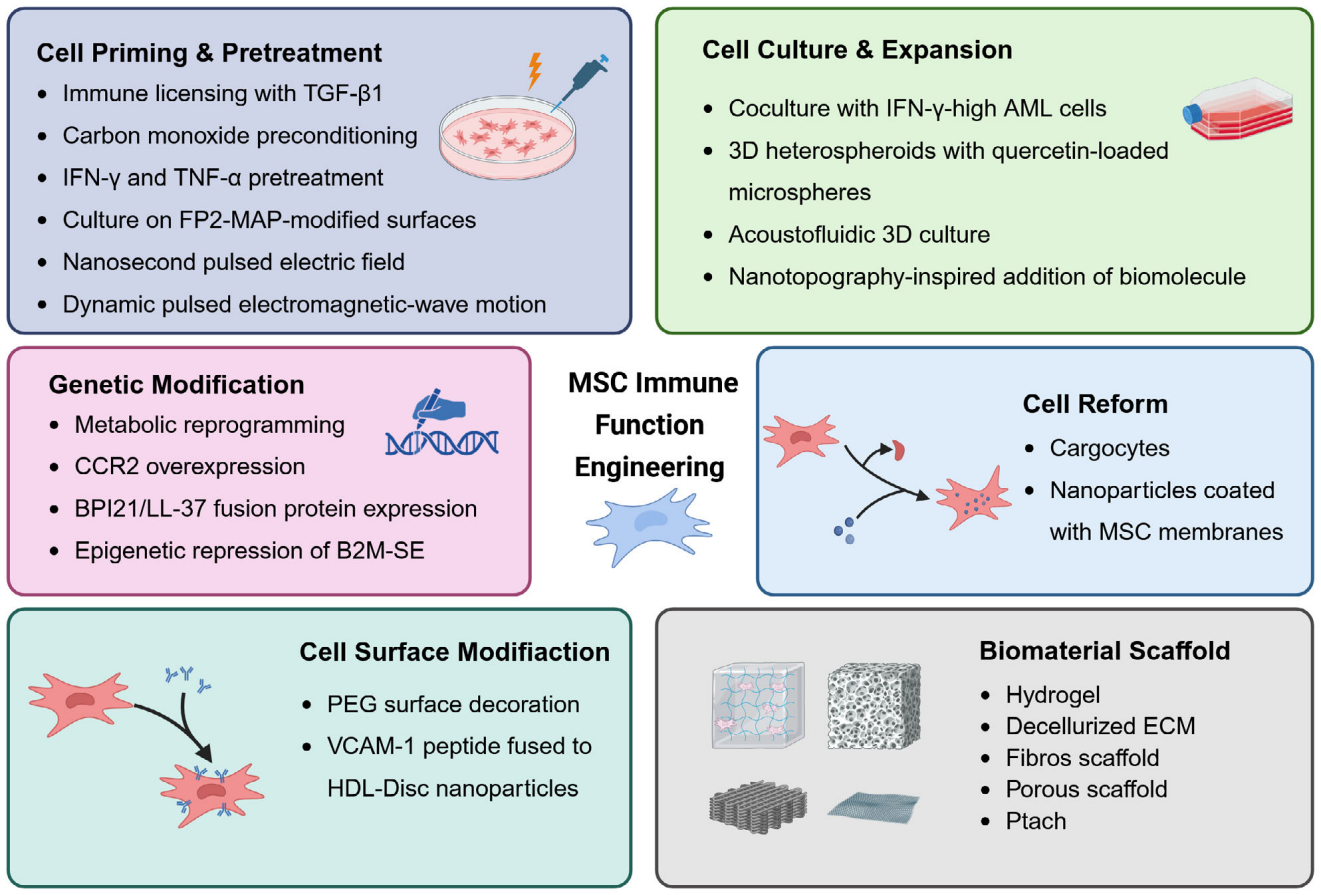
MSC‐based engineering. Schematic diagram of engineering and facilitative approaches to modify MSC immunomodulation abilities as discussed in the review. Various approaches have been developed and utilized to make MSCs more applicable for clinical scenarios.

There is a general consensus that the intrinsic biological potential of MSCs alone is insufficient to overcome critical translational hurdles such as poor in vivo survival, limited retention, and functional variability [98, 117, 118, 119]. By integrating insights from materials science, molecular biology, genetic biology, and synthetic biology, researchers are developing significantly more advanced approaches to mitigate these inherent limitations to enhance the therapeutic efficacy of MSCs. This section provides an overview of these MSC engineering strategies (Figure 4 and Table 4).

**TABLE 4:** MSC engineering approaches and resultant immunosuppression performance.

Source of MSCs	Approach	Animal model	Immune‐related influencer/performance	References
Human AD‐MSCs	Pretreatment with IFN‐γ and TNF‐α	Colitis mouse	Upregulated SOD2 enhances the immunomodulation ability at the cost of adipose differentiation	[99]
Human BM‐MSCs	Carbon monoxide preconditioning	Sepsis mouse	Induced MSCs autophagy activation enhances the paracrine function	[100]
Mouse BM‐MSCs	Licensing with TGF‐β1	Corneal allografted mouse	Enhanced PD‐L1, CD73, and PGE2 secretion, in turn promote immunomodulation ability	[120]
Human/mice MSCs	Metabolic reprogramming	GvHD mouse	AMPK/glycolytic switch mediated suppression of pro‐inflammatory T cells	[121]
Human BM‐MSCs	Dynamic pulsed electromagnetic‐wave motion	Not specified	Induce ROS‐scavenging genes (GPX1b1, GPX1b2, TXNR1, NOX4, CAT, SOD2) expression	[122]
Human BM‐MSCs	Conditioned medium from pulsed electromagnetic fields (PEFs)‐treated MSCs	NA	Enhanced paracrine function elevates the BMP2, BMP4, TSP‐2, IL‐1RA, and IL‐10 secretion	[123]
Rat/human BM‐MSCs	Nanosecond pulsed electric fields (nsPEFs) pretreatment	OA rat	Suppressed IL‐1β and MMP13 in OA chondrocytes, rescuing their OA phenotype	[124]
Human UC‐MSCs	CEACAM1‐4S isoform overexpression	GvHD mouse	Elevated CEACAM1 inhibits T cells activation and proliferation as well as monocytes inflammatory response	[32]
Human UC‐MSCs	CCR2 overexpression	Diabetic wound mouse	CCR2 mediates higher expression of IL‐6, TNF‐α and promote Treg recruitment	[125]
Human UC‐MSCs	BPI21/LL‐37 fusion protein expression	Sepsis mouse	The fusion protein enhances antibacterial targeting of the engineered MSCs	[101]
Mouse BM‐MSCs	CRISPR‐dCAS9‐mediated RelA inhibition + Sox9 activation	OA mouse	Inhibited RelA and activated Sox9 reduce the local inflammation	[44]
Human ES‐derived MSCs	Epigenetic repression of B2M‐SE (β2m super enhancer)	NA	Reduced HLA‐I expression consequently reduces the immunogenicity of the cells	[126]
Mouse BM‐MSCs	NLRP3 inflammasome deletion	Colitis mouse	Suppressed IL‐10 secretion	[127]
Mouse MSCs	DNA template‐directed polyvalent antibody synthesis on MSCs surface	Colitis mouse	Improved adhesion to inflamed endothelium of MSCs which can facilitate cell homing	[102]
Human WJ‐MSCs	Culture on FP2‐MAP‐modified surfaces (EDC/NHS chemistry)	Osteoarthritis mouse model	Enhanced anti‐inflammatory gene IL‐1RA, IL‐10, TIMP2 expression	[103]
Mouse BM‐MSCs	Coculture with M1 macrophages	Abortion mouse	Enhanced expression of TSG‐6 and CD200 promote the anti‐inflammatory phenotype of macrophage	[105]
Human BM‐MSCs	Coculture with IFN‐γ‐high AML cells	AML mouse	Enhanced IDO1expression promotes Treg induction	[128]
Human AD‐MSCs	3D heterospheroids with quercetin‐loaded microspheres	Colitis mouse	Suppressed T helper cell polarization toward Th1/Th17	[108]
Human BM‐MSCs	Acoustofluidic 3D culture	NA	Enhance anti‐inflammatory MSCs secretome	[129]
Human BM‐MSCs	Nanotopography‐inspired addition of biomolecule	NA	Maintained immune properties during large‐scale expansion.	[106]
Human AD‐MSCs	VCAM‐1 peptide fused to HDL‐Disc nanoparticles (membrane fusogenicity technology)	Alzheimer's disease mouse	4F peptides mediated targeting enhance the cell homing toward inflammatory endothelium	[130]
C3H10T1/2 cells (mouse MSCs)	PEG surface decoration	Liver injury mouse	The surface modification improves targeting ability to injured liver	[131]
Human AD‐MSCs	Cargocytes (enucleated MSCs engineered with CXCR4/CCR2 and IL‐10 delivery)	Acute pancreatitis mouse	Targeted IL‐10 delivery attenuates injury site inflammation.	[132]
Rat BM‐MSCs	Nanoparticles coated with MSC membranes	MCAO (stroke) mouse	Promote ROS scavenging to attenuate the inflammatory environment	[133]
Rat BM‐MSCs	VCAM‐1 and dopamine decoration	Myocardial infraction (MI) rat	Suppressing HB‐EGF–EGFR signaling between anti‐inflammatory macrophages and activated fibrillates	[134]

### 3.1 Cell Priming

Preconditioning strategies seek to enhance the inherent immunomodulatory capabilities of MSCs by exposing them to specific stimuli or conditions, including pharmacological agents, cytokines, or environmental factors. The process of preconditioning induces a state of readiness in MSCs, enabling them to respond more effectively to inflammatory signals and augment their immunomodulatory functions. For instance, preconditioning human BM‐MSCs with carbon monoxide increases autophagy through elevated expression of microtubule‐associated protein 1A/1B light chain 3B (LC3B), Beclin1, autophagy protein 5 (ATG5), and ATG12. This enhancement leads to increased paracrine secretions containing specific microRNAs such as miR‐145 and miR‐193a, which promote neutrophil phagocytosis and macrophage efferocytosis. Experimentally, this process improves the survival of human BM‐MSCs in murine sepsis models [100]. Similarly, treatment of murine BM‐MSCs with transforming growth factor‐β 1 (TGF‐β1) enhances their ability to modulate inflammatory macrophages. This effect correlates with elevated expression of immunosuppressive markers, including programmed cell death ligand 1 (PD‐L1) and CD73, and increased PGE2 production, which is crucial for Treg expansion and T cell activation, showing profound efficacy in corneal allografted mice [120]. Another innovative strategy involves reprogramming MSC metabolism using agents like oligomycin or 2‐deoxy‐D‐glucose (2DG). Oligomycin induces an AMPK‐dependent glycolytic switch in both human and mouse MSCs, consequently suppressing pro‐inflammatory T cells and enhancing their immunomodulatory effects in conditions like delayed‐type hypersensitivity (DTH) and GVHD. In contrast, 2DG‐modified MSCs exhibit attenuated immune regulatory functions [121]. Interestingly, the immunomodulatory enhancement of MSCs may occur at the expense of their differentiation potential. Upon treatment of human adipose‐derived MSCs (hAD‐MSCs) with interferon γ (IFN‐γ) and tumor necrosis factor α (TNF‐α), there is an observed elevation in superoxide dismutase 2 (SOD2) levels. This upregulation of SOD2 inhibits adipocyte differentiation, thereby augmenting the immunosuppressive capabilities of MSCs in mouse with induced colitis [99].

External physical stimuli also play a significant role in altering MSC phenotype and function. Pulsed electromagnetic fields (PEFs) are one of the widely used physical stimuli that are capable of initiating healing processes. Notably, different PEF parameters (e.g., field strength, pulse duration) diversely affect cellular phenotypes, including differentiation, immunomodulation, and proliferation capacities [135, 136]. For instance, hBM‐MSCs exposed to dynamic pulsed electromagnetic‐wave motion exhibit upregulation of reactive oxygen species (ROS) scavenging‐related genes, including glutathione peroxidase b1 isoform (GPX1b1), GPX1b2, thioredoxin reductase 1 (TXNR1), NADPH oxidase 4 (NOX4), catalase (CAT), and SOD2, while concurrently promoting cell proliferation [122]. Conditioned medium obtained from PEFs‐treated hBM‐MSCs has demonstrated the ability to reduce nitric oxide synthases (NOS), IL‐6, Matrix metallopeptidase 13 (MMP13), and cyclooxygenase‐2 (COX2) levels in inflammatory environment. Meanwhile, the treatment also showed profound ability in increasing local chondrocyte proliferation and migration, promoting cartilage regeneration in the reduced inflammatory environment [123]. Furthermore, nanosecond PEFs pretreatment has been applied to both rat and human BM‐MSCs, where they effectively decrease IL‐1β and MMP13 levels in co‐cultured OA chondrocytes in rat models while exhibiting a promoted chondrogenic differentiation phenotype [124].

### 3.2 Genetic Modification

Extensive research has demonstrated that certain key genes and pathways are crucial for the immune regulatory functions of MSCs in treating different diseases, including GVHD, diabetic wounds, and OA, indicating that genetic modifications of MSCs can be highly effective in enhancing the potential therapeutic effect.

Controlling specific gene expression through gene transduction and editing techniques is an effective method for modulating the immunomodulatory capacity of MSCs. Umbilical cord mesenchymal stromal cells (UC‐MSCs) engineered to express the carcinoembryonic antigen‐related cell adhesion molecule 1 (CEACAM1)‐4S isoform, where CEACAM1 is a highly conserved as a immune evasive factor, exhibited significantly improved suppression of T cell activity and enhanced survival in mouse models of GVHD [32]. C–C motif chemokine ligand 2 (CCL2) is found highly expressed in diabetic wound site. Overexpression of C‐C chemokine receptor type 2 (CCR2), a CCL2 receptor, in human UC‐MSCs significantly enhanced targeting to diabetic wound sites, reduced the levels of inflammatory cytokine IL6 and TNF‐α, inhibited macrophage infiltration, and prompted Treg cell recruitment in mouse models. This attenuated inflammatory response significantly accelerated diabetic wound healing [125]. Additionally, modifying hUC‐MSCs to express antibacterial fusion peptides (BPI21 and LL‐37) broadened their ability to suppress inflammatory cytokines while promoting IL‐10, as evidenced in the sepsis model [101]. By utilizing CRISPR‐dCas9‐mediated inhibition of a TNF/ NF‐κB activator v‐rel avian reticuloendotheliosis viral oncogene homolog A (RelA) in mouse BM‐MSCs, modified cells not only inhibit proinflammatory cytokines in splenocytes but also halt phytohemagglutinin M‐induced proliferation. Combined with SRY‐Box transcription factor 9 (Sox9) activation, these modified cells demonstrated significant efficacy in OA treatment by inhibiting the inflammatory milieu and promoting chondrogenesis in mouse models [44]. Wang et al. developed a novel strategy to overcome immune rejection in allogeneic cell therapies via targeted epigenetic repression of the β2‐microglobulin super‐enhancer (B2M‐SE) in human MSCs. This method effectively reduces the surface expression of β2m, a key component of HLA‐I, to levels that prevented allogeneic T‐cell activation while retaining sufficient expression to evade natural killer (NK) cell‐mediated cytotoxicity [126]. In addition, elevating the expression of NOD‐like receptor pyrin domain‐containing protein 3 (NLRP3) inflammasome, which can trigger glucose transporter 1 (Glut1), in mouse BM‐MSCs enhanced osteogenic differentiation without altering surface marker profiles and improved therapeutic efficacy in inflammatory conditions such as colitis, evidenced by increased IL‐10 production [127]. An innovative method, involved synthesizing polyvalent antibodies directly on the mouse MSCs surface through DNA template‐directed biomolecule assembly, significantly improved adhesion of MSCs to inflamed endothelium and has shown efficacy in both in vitro (inflamed endothelium) and in vivo (colitis mouse) models of acute inflammation and inflammatory bowel disease [102]. Collectively, these examples suggest that targeted genetic interventions can optimize MSC functionality for specific therapeutic application.

### 3.3 Cell Culture and Expansion

Cell expansion represents a critical component of MSC therapy, as effective clinical applications require substantial cell quantities. As the fraction of MSCs isolatable from tissues is inherently limited, extensive in vitro expansion is necessitated to fulfill therapeutic doses in vitro [137].

Traditional in vitro expansion methods often compromise immunomodulatory capacity. Therefore, optimizing culture conditions is essential to enhance MSC proliferation while maintaining their immunomodulatory properties and therapeutic potential. For instance, by utilizing N‐ethyl‐N′‐(3‐(dimethylamino)propyl)carbodiimide/N‐hydroxysuccinimide (EDC/NHS) chemistry, researchers have successfully fused FGF‐2‐derived peptide (FP2) to immobilize mussel adhesive proteins (MAP) on culture plates. Human Wharton's jelly‐derived MSCs (hWJ‐MSCs) cultured on these modified surfaces exhibited enhanced in vitro proliferation and differentiation ability, along with in vivo immunomodulatory capacities, as evidenced by studies conducted in mouse OA models [103]. Additionally, employing nanotopography to control intracellular tension and promote oxidative glycolysis during culture has been shown to maintain the immune properties of Stro‐1 positive hBM‐MSCs during large‐scale production [106].

Coculture with inflammatory cells represents another effective approach to modify MSC function. Direct interaction with M1 macrophages has been shown to increase the expression of tumor necrosis factor‐inducible gene 6 protein (TSG‐6), a factor that enhances the immunosuppressive properties of mouse BM‐MSCs by reducing inflammatory cytokine production and T cell proliferation [105]. This demonstrates the dynamic adaptability of MSCs to inflammatory microenvironments. Furthermore, coculture with IFN‐γ high acute myeloid leukemia (AML) cells induces hBM‐MSCs to upregulate genes associated with Treg cell recruitment and differentiation. This process enhanced their ability to induce Tregs via IDO1, effectively skewing MSCs toward an immunosuppressive phenotype in mouse AML models. Such adaptations are crucial for developing MSC‐based therapies aimed at modulating immune responses in various pathological conditions [128].

Moreover, three‐dimensional (3D) culture systems have shown promise in strengthening the immunomodulation of MSCs. For example, by culturing hAD‐MSCs in heterospheroids with quercetin‐loaded microspheres, researchers observed that MSCs could suppress T helper cell 1 (Th1)/Th17 differentiation while promoting regulatory T cells and anti‐inflammatory macrophages in murine colitis. Concurrently, the MSC‐loaded heterospheroids secreted the paracrine factor, PGE2, which significantly promoted epithelial regeneration [108]. The use of acoustofluidic techniques on hBM‐MSCs could enhance the secretome during 3D culture, leading to increased production of anti‐inflammatory cytokines in response to IFN‐γ, thereby regulating inflammation effectively [129].

In summary, advancements in MSC expansion techniques, coculture strategies, and 3D culture systems are essential for optimizing the immunomodulatory properties of MSCs and enhancing their therapeutic potential in clinical applications.

### 3.4 Cell Surface Modification

Site‐specific delivery is another vital aspect of MSC‐based therapies, often achieved through surface modification techniques that enable recognition of specific cellular or molecular targets. A promising strategy utilizes membrane fusogenic technology. By fusing vascular cell adhesion molecule 1 (VCAM‐1) specialized VBP peptide, a vascular cell adhesion molecule 1 specialized peptide, with recombinant high‐density lipoprotein (HDL)‐Disc nanoparticles containing APOs‐mimicking peptides (4F), researchers engineered a biobridge that effectively transferred VBP onto the MSC membrane. This technique yielded a remarkable 3.86‐fold increase in the linkage between MSC and inflammatory endothelium compared to traditional methods. The modified AD‐MSCs thereby acquired enhanced ability to recognize and adhere to inflammatory endothelium, facilitating targeted reduction of neuroinflammation in Alzheimer's disease models [130]. Similarly, by decorating rat BM‐MSCs with both VCAM‐1 and dopamine enabled dual functionality: VCAM‐1 mediated recruitment to inflammatory sites, while dopamine promoted local ROS clearance and immune response suppression. This synergy significantly enhanced MSC targeting efficiency and survival in vivo, demonstrating substantial protection against cardiac fibrosis in myocardial infarction models [134]. In a case of liver regeneration, surface modification of C3H10T1/2 cells (an MSC‐like cell line) with polyethylene glycol (PEG) augmented their adhesion capacity to mouse aortic endothelial cells. The PEG‐modified cells exhibited refined homing capability to injured liver tissue, subsequently reducing leukocyte infiltration at the damage site and accelerating liver regeneration [131]. Cell coating is another approach used for improving homing ability. BM‐MSCs coated with pericellular collagen I showed great potential in facilitating cell targeting and retention, while promoting cartilage regeneration, in rabbit damaged joint with no inflammatory response found [138].

### 3.5 Cell Reform

The immunosuppressive properties of MSCs can also be harnessed to facilitate the delivery of therapeutic agents. Bioengineered “cargocytes”—enucleated cells derived from immortalized hAD‐MSCs—retain critical cellular functions while serving as targeted delivery vehicles. Cargocytes exhibit enhanced deformability, allowing them to navigate through small capillaries and endothelial layers more efficiently than their parental MSCs. These cells can be genetically engineered to express chemoattractant receptors, such as C‐X‐C chemokine receptor type 4 (CXCR4) and CCR2, promoting chemotaxis and homing to inflamed tissues. Studies demonstrated that cargocytes effectively delivered bioactive cytokines, such as human IL‐10, to attenuate tissue inflammation and damage in mice with acute pancreatitis [132]. Similarly, nanoparticles coated with rat BM‐MSC membranes served as camouflage for targeted delivery to cerebral ischemic penumbra, providing ROS scavenging and neuroprotection in middle cerebral artery occlusion (MCAO) mouse models. This innovative approach highlights the versatility of MSC engineering in enhancing therapeutic outcomes [133].

Beyond creating a regenerative‐friendly environment, engineering strategies enhance MSC‐mediated tissue repair by optimizing cell delivery, migration, and differentiation [139, 140]. One significant strategy involves UC‐MSC surface modification with Dimyristoylphosphatidylethanolamine (DMPE)‐PEG‐pPB (cyclic peptide pPB, C∗SRNLIDC∗) conjugates. This modification enables targeted homing to activated hepatic stellate cells without functional compromise, while facilitating mitochondrial transfer from MSCs to injured cells, promoting regeneration and mitigating cellular damage [141]. Additionally, the differentiation ability of MSCs can be optimized for specific conditions. Gene therapy approaches can transduce MSCs with genes for controlled protein expression, such as lasting expression of bone morphogenetic protein 2 (BMP2) [139, 142] or decreased expression of nuclear factor erythroid 2 related factor 2 (Nrf2) [143], which induces osteoblast differentiation and attracts host cells for bone matrix formation, overcoming the short half‐life of growth factors. Application of nanosecond PEFs further potentiates differentiation capacity by enhancing the trilineage differentiation potential of MSCs by temporarily downregulating DNA methyltransferase 1 (DNMT1) and consequently elevating expression of pluripotency genes like octamer‐binding transcription factor 4 (OCT4) and NANOG [144]. The regenerative ability of MSCs itself should not be neglected during tissue regeneration, although the immune‐regulatory function of MSCs is emphasized.

## 4 Biomaterial‐Based Immune Regulation of MSCs

While MSC‐based therapies hold significant promise for regenerative medicine, some of their inherent limitations can be addressed by the application of scaffolds. One of the primary challenges in MSC‐based therapies is the low retention and survival rates of transplanted cells, which significantly hinder their therapeutic efficacy. Scaffolds, particularly those made from advanced biomaterials, offer a solution by providing a supportive microenvironment that enhances cell retention, viability, and integration into host tissues [145, 146]. This enhanced microenvironment is crucial for maximizing the therapeutic effects of MSCs, especially in challenging conditions such as tissue injury and inflammation. In both bone and spinal cord injuries, biomaterial‐based scaffolds can significantly maintain MSCs survival and engraftment, thus benefiting MSC‐mediated inflammatory environment inhibition and in situ repair [147, 148]. Injectable hydrogels represent a particularly versatile scaffold modality, enabling minimally invasive delivery to anatomically complex sites while further promoting MSC survival and integration [148]. The scaffold‐MSC synergy not only overcomes transplantation barriers but amplifies therapeutic outcomes across diverse applications [118, 149]. To further optimize MSC functionality and therapeutic efficacy, researchers have investigated diverse scaffold materials such as hydrogels, porous scaffolds, electrospun fibers, micropatterned biomaterials, and decellularized scaffolds, each designed to create a supportive microenvironment for MSCs (Table 5).

**TABLE 5:** Immunosuppression performance of MSC‐scaffold composites.

Source of MSCs	Material	Animal model	Immune‐related influencer	Outcome	References
Human UC‐MSCs	Gel‐MA and HA‐MA hydrogel microcapsules	Bleomycin‐induced pulmonary fibrosis mice	Activated MAPK/MMP pathways, ameliorated the inflammatory microenvironment	Decreased the accumulation of fibrillar collagen within the lung parenchyma; preserved normal architecture of the lungs	[150]
Rat BM‐MSCs	Agarose hydrogel	Traumatic brain injury rat	Activated Fas/FasL pathway, reduced the host cytotoxic CD8+ T cell population	Decreased injury cavity	[151]
Rat MSCs	Thermosensitive hydroxypropyl chitin hydrogel	Calvarial defect rat	Promoted M2 polarization	Promoted vascularization and osteoinduction	[152]
Rat BM‐MSCs	Collagen hydrogels	No animal model used	Activated PI3K‐Akt pathway, suppressed LPS‐induced inflammatory reaction	Promoted neuroprotection and neurogenesis via PI3K‐Akt pathway	[153]
Rat AD‐MSCs	Electrospun scaffolds	Skin excisional wound rat	Promote MSCs to secrete anti‐inflammatory factors. Promoted the macrophage recruitment and enhanced the polarization of macrophages toward the pro‐healing phenotype	Promoted wound closure and improved collagen deposition	[154]
UC‐MSCs	Alginate microcapsules	Skin excisional wound mice	Suppressed the NfκB pathway activation of macrophages in response to LPS	Reduced inflammation response in the engrafted site	[155]
Rat BM‐MSCs	Low‐temperature deposition modeling printed sponge‐like scaffold	Distal femoral defect rat	Enhanced the secretion of immunomodulatory factors by MSCs and promoted M2 macrophage polarization	Promoted osteogenesis and angiogenesis	[156]
Human BM‐MSCs	Hierarchical intrafibrillarly mineralized collagen	Critical‐sized bone defects rat	Facilitated M2 macrophage polarization and interleukin (IL)‐4 secretion	Promoted endogenous bone regeneration	[157]
Human UC‐MSCs	Hyaluronic acid scaffold	SCI rat	Promoted the transient release of a significant amount of IL‐10 by MSCs	Accelerated functional recovery of spinal cord	[158]
Mice BM‐MSCs	Bone bioceramic scaffold	Calvarial defect mice	Activated anti‐inflammatory M2 macrophages. Enhanced BMP‐Smad, Oncostatin M (OSM), and Wnt/β‐catenin pathway	Improved osteogenesis and bone quality	[159]
Mice BM‐MSCs	Mineral‐particles‐coated gelatin microribbon	Calvarial defect mice	Promoted M2 polarization	Increased osteogenesis and bone volume	[160]
Human BM‐MSCs	Cartilage‐derived matrix	No animal model used	Inhibited IL‐1	Improved ECM deposition	[161]
Human BM‐MSCs	Electrospun polycaprolactone/silk fibroin (PCL/SF) composite fibrous scaffold	Rat tendon adhesion model	Promoted M2 polarization and inhibited various inflammatory cytokines	Reduced foreign‐body reaction and alleviated tendon adhesion	[162]
Human UC‐MSCs	3D‐printed poly(lactide‐co‐glycolide) scaffolds	Heterotopic transplantation model of SCID mice	Increased the migration of M2 macrophages and the repolarization of M1 macrophages to M2 phenotype by TGFβI pathway	Promoted osteogenesis	[163]
NA	Microvascular hydrogel patch	Cutaneous injury mice	promote M2 polarization in wound tissue	Accelerated wound tissue healing and improved ECM deposition	[164]
Human ESC‐derived MSCs	Porous microneedles patch	SCI rat	Reduced expression of IL‐1β and TNF‐α; and elevated expression of the TGF‐β and Arg‐2	Improved hindlimb locomotor functional recovery and muscle control	[165]

Hydrogels, recognized for their tunable physical properties, biocompatibility, and biodegradability [166], significantly enhance MSC immunomodulation by establishing a protective microenvironment. For instance, human UC‐MSCs microencapsulated in photocrosslinkable methacrylated gelatin (Gel‐MA) and methacrylated hyaluronic acid (HA‐MA) hydrogels mitigated pulmonary fibrosis in mice via mitogen‐activated protein kinase (MAPK)/MMP pathway‐mediated extracellular matrix (ECM) remodeling, reducing fibrotic markers and restoring lung architecture [150]. Similarly, immunosuppressive agarose hydrogels with Fas ligand (FasL) releasing lipid microtubes improved the survival of allogeneic BM‐MSCs in traumatic brain injury (TBI) models by suppressing Fas/FasL‐mediated immune rejection and reducing host macrophage activation. Subsequently, improved MSC retention reduced injury cavity volume in TBI [151]. Thermoresponsive hydroxypropyl chitin hydrogels and 3D‐printed poly(ε‐caprolactone)/nano‐hydroxyapatite scaffolds promoted macrophage polarization from pro‐inflammatory M1 to anti‐inflammatory M2 in critical‐sized bone defects, while enhancing local vascularization and osteogenesis of inherent BMSCs, synergizing tissue regeneration and inflammation reduction [152]. Collagen hydrogels loaded with BM‐MSC spheroids further inhibited inflammatory cytokines (TNF‐α and PGE2) via phosphatidylinositol 3‐kinase (PI3K)‐protein kinase B (Akt) signaling. This dual‐function construct provided structural support while creating a favorable microenvironment that elevated neural stem cell neurogenesis [153]. Collectively, these systems highlight the capacity of hydrogels to shield MSCs while actively reprogramming local immune responses toward repair. However, the intrinsic limitations of hydrogels often include weak mechanical properties and slow response to environmental stimulation, along with poor biocompatibility for undegradable hydrogels [167].

Porous scaffolds amplify MSC immunomodulation through structural permeability and prolonged cell residence at injury sites. By utilizing low‐temperature deposition modeling, a porous scaffold was built using copolymer poly (L‐lactic acid‐ε‐caprolactone) PLCL and hydroxyapatite nanoparticles for improved in situ adhesion and proliferation of mouse BM‐MSCs. These scaffolds concurrently elevated secretion of immunomodulatory factors (COX2, PGE2, TSG6), driving vascularized bone regeneration in rat femoral defect models [156]. Porous hyaluronic acid (HA) scaffolds promoted site adhesion and growth of hUC‐MSCs, as seen in spinal cord injury (SCI) models where encapsulated MSCs release IL‐10 to dampen inflammation [158]. Hierarchical intrafibrillar‐mineralized collagen scaffolds mimicking bone microstructure recruited endogenous MSCs and M2 macrophages. When loaded with interleukin‐4 (IL‐4), these scaffolds significantly improved critical‐sized bone defect healing in rats [157]. Electrospun nanofibrous scaffolds represent a type of highly porous scaffold manufactured by the electrospinning process. The varying pore size and the high surface area‐to‐volume ratio of electrospun fibrous scaffolds provide a preferable environment for cell growth and adhesion [168]. Fiber orientation (random/aligned/mesh) in polycaprolactone scaffolds directly modulated AD‐MSC cytokine secretion, with mesh configurations maximizing anti‐inflammatory factors (PGE2, hepatocyte growth factor (HGF)) and inducible nitric oxide synthase (iNOS), which resulted in superior anti‐inflammatory responses in vitro and accelerated wound healing in rat excisional models in vivo [154]. Analogously, alginate microcapsules encapsulating UC‐MSC secretome achieved controlled release at implantation sites, reducing TNF‐α, IL‐6, and nuclear factor kappa‐B (NF‐κB) levels to polarize macrophages toward the M2 phenotype. An alginate scaffold containing MSC secretome diminished fibrotic encapsulation at the scaffold injection site in adult mice compared with the scaffold only [155]. In the disgin of porous scaffolds, while mechanical properties, biocompatibility, and degradation kinetics remain critial considerations [169], permeability, which is highly associated with engrafted cell bioactivity [170], should also be taken into consideration.

Micropatterned biomaterials enable precise spatial orchestration of cellular interactions. When co‐cultured with BM‐MSCs and macrophages, these substrates couple osteogenesis with NF‐κB suppression and M2 polarization in calvarial defects, simultaneously enhancing MSC‐mediated bone formation [159]. Micro‐sized tricalcium phosphate (mTCP) coatings of porous gelatin microribbon scaffolds optimized BM‐MSC‐mediated M2 macrophage polarization in mouse 3.5 mm cranial defects. This effect was amplified by aspirin‐loaded scaffolds that concomitantly inhibited M1 polarization and osteoclast activation. With inhibited bone resorption coupled to MSC‐mediated osteogenesis, the complex exhibited profound bone healing ability in bone defects [160].

Decellularized scaffolds leverage native tissue architecture to potentiate immunomodulation. A cartilage matrix engineered with a lentiviral IL‐1RA delivery system utilized hBM‐MSC immunomodulation and differentiation, reducing MMP activity, promoting M2 macrophage polarization, inhibiting B cell differentiation, and maintaining the in situ chondrogenesis and osteogenesis level [161]. The ECM, a core component of decellularized scaffolds, further amplifies MSC immunosuppression when integrated into synthetic scaffolds. For instance, hBM‐MSC‐derived ECM combined with electrospun fibers reduced foreign body response (FBR) and enhanced bone regeneration in rats through polarizing macrophages to M2 [162]. Similarly, PLGA scaffolds modified with hUC‐MSC‐derived ECM increased M2 activity via TGF‐β1 in mice, mitigating FBR and improving scaffold integration while initiating local osteogenesis [163]. These approaches underscore the dual role of scaffold material and ECM interactions in shifting macrophages from M1 to M2, optimizing tissue repair through structural and immune‐modulatory synergy. The concerns induced by decellularized scaffolds include, but not limited to, the potential immunogenicity of the scaffold, the deleterious residue of the product, and the quality control of the manufacturing protocol [171].

Beyond these scaffold materials, the patch itself offers a less invasive delivery approach for MSCs, significantly enhancing the efficiency and ease of their in situ implantation [172]. MSC‐containing GelMA hydrogel patch with embedded micro‐vesicles is found to promote M2 polarization in wound tissue of mouse [164]. Meanwhile, patches are frequently combined with microneedles to improve targeting and efficacy. Patches covered with MSCs derived from human ESCs, along with microneedles delivering exosomes, have demonstrated inflammation‐suppressive effects in rat spinal cord injury [165].

However, caution is warranted as certain bioactive scaffolds may inadvertently amplify inflammatory responses, thereby compromising MSC immunomodulatory functions. It was found that bioactive hydroxyapatite scaffold could upregulate key inflammatory factors such as TNF and ILs via triggering receptor expressed on myeloid cells 1 (TERM1). This pro‐inflammatory cascade counteracts MSC immunosuppression and ultimately impedes tissue regeneration [173].

It should be noted that biomaterials and scaffolds are primarily utilized to enhance MSC regenerative capabilities—rather than direct immunomodulation—by modulating differentiation and proliferation [174, 175, 176]. Softer photocrosslinked 3D gelatin scaffolds significantly enhanced cell spreading and adipogenesis of encapsulated human BM‐MSCs compared to stiffer hydrogels by decreasing cellular caveolin‐1 expression and altering plasma membrane fluidity [177]. Incorporating graphene oxide (GO) nanosheets into gelatin methacrylate (GelMA) scaffolds enhanced osteogenic differentiation of encapsulated hMSCs via BMP signaling pathways [178]. Similarly, using a photocross‐linked hydrogel containing allogeneic AD‐MSCs and autologous platelet‐rich plasma (PRP) significantly improved the regeneration of focal osteochondral defects in a rabbit model [179]. Injectable porous microspheres (PMs) composed of aldehyde‐modified poly(lactic‐co‐glycolic acid) can effectively recruit endogenous MSCs, while dual loading with platelet‐derived growth factor‐AB (PDGF‐AB) and kartogenin enhanced chondrogenic differentiation [180].

These findings demonstrate that scaffold modifications, including ECM integration, can leverage complementary biomaterial properties to create permissive microenvironments for MSC functions. Critically, scaffolds do not merely provide passive support but actively synergize with MSCs through reciprocal interactions: enhancing immunomodulatory functions while simultaneously potentiating regenerative capacity. The synergistic interplay between these components amplifies therapeutic outcomes, exceeding the efficacy achievable by either approach in isolation.

## 5 Current Research Development on Extracellular Vesicle Engineering

**FIGURE 5:**
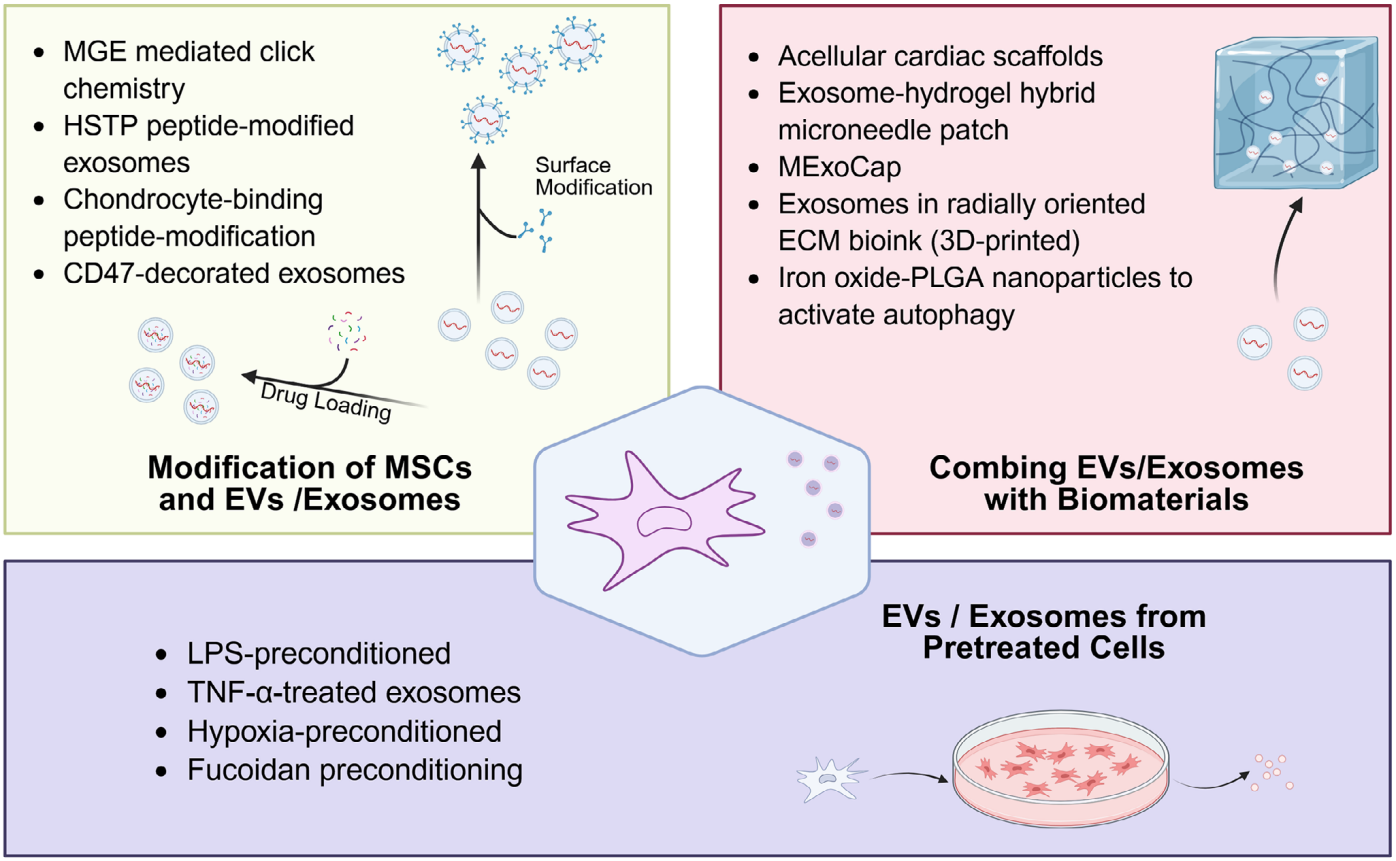
MSC‐derived exosome engineering. Schematic diagram of engineering approaches to utilize the immunomodulation function of MSC‐derived EVs. In addition to cell pretreatment and scaffold delivery, EVs are also taken advantage of their acellular properties.

Extracellular vesicles (EVs) are nano‐sized particles released by cells that facilitate intercellular communication through the transfer of proteins, lipids, and nucleic acids [181]. Among these EVs, exosomes (30–150 nm diameter) play crucial roles in inflammation, immune responses, and various diseases and treatments [182, 183]. MSC‐derived exosomes have been found to recapitulate the therapeutic benefits of parental MSCs while circumventing risks associated with whole‐cell therapies. This section focuses on engineering strategies to enhance the immunomodulatory properties of MSC‐derived EVs and exosomes (Figure 5).

MSC‐derived exosomes can significantly influence immune cell functions and key inflammatory pathways, positioning them as a promising candidate agent for treating immune disorders. Exosome released from MSCs often carries key biomolecules, where miRNAs (e.g. miR‐27b‐3p [184], miR‐100‐5p [185]) are more often reported. The biomolecules are uptaken by cells in the microenvironment, contributing to immune regulation and tissue regeneration [186, 187]. Multiple diseases have been proven to be alleviated by MSC‐derived exosomes in animal models, including GVHD, fibrosis, RA, and OA (Table 6).

**TABLE 6:** Immunosuppression performance of MSC‐derived EVs/exosomes.

Source of exosomes	Approach	Animal model	Immune‐related influencer/performance	References
Rat BM‐MSCs	LPS‐preconditioned exosomes	Skin grafted mouse	Promoted M2 polarization and suppressed NF‐κB/NLRP3 signaling‐meditated M1 polarization of macrophage	[188]
Human G‐MSCs	TNF‐α‐treated exosomes	Periodontitis mouse	miR‐1260b mediates M2 polarization of macrophage	[15]
Human G‐MSCs	TNF‐α‐treated exosomes	IRI mouse	miR‐21a‐5p binds with apoptosis‐related gene PDCD4, consequently regulate neuroinflammation and cell death	[16]
Rat BM‐MSCs	Fucoidan‐preconditioned exosomes	OA rat	miR‐146b‐5p can suppress TRAF6 activation, which then regulates inflammatory response	[189]
Human AD‐MSCs	Tangential flow filtration for exosome isolation, MGE mediated click chemistry	CIA mouse	Enhance EV macrophage affinity via surface modification	[190]
Mouse BM‐MSCs	Low‐intensity pulsed ultrasound stimulation	NA	Suppressed MAPK by miR‐328‐5p and miR‐487b‐3p	[191]
Mouse BM‐MSCs	Exosomes encapsulated in PLGA microcapsules (MExoCap)	Renal ischemia‐reperfusion injury (RIRI) mice	The microcapsules facilitate the sustained exosome release of EV	[192]
MSCs (3D cultured)	Exosome‐hydrogel hybrid microneedle patch	Spinal cord injury rat	The patch enables sustainable in situ exosome release	[193]
Human BM‐MSCs	Exosomes in radially oriented ECM bioink (3D‐printed)	Cartilage defect rabbit	Promote M2 macrophage polarization; Rescue mitochondria activity	[194]
Porcine cardiac adipose tissue‐derived MSCs	Acellular cardiac scaffolds	Myocardial infarction pig model	Reduced macrophage and T cell infiltration in the injury site	[195]
Mouse BM‐MSCs	Exosome isolation	HSCT mouse	Promoted M2 macrophage polarization and enhanced Treg cell differentiation	[196]
Human BM‐MSCs	Exosome isolation	cGVHD mice	Suppressed Th17 cells, inhibited the activation and infiltration of CD4+ T cells, and induced Treg	[197]
Human placental MSCs	Exosome isolation	Mdr2^−/−^ mouse	Inhibited Th 17 cells cell differentiation through IκBζ downregulation	[198]
Human UC‐MSCs	Exosome isolation	CCl4‐induced mouse	miR‐27b‐3p suppresses YAP/LOXL2 pathway, and consequently alleviates liver fibrosis	[184]
Human UC‐MSCs	Exosome isolation	Skin injury mouse	Modulated Nrf2 pathway mitigates oxidative stress‐related inflammation	[199]
Human ES‐MSCs	Exosome isolation	Temporomandibular joint OA rats	Suppressed IL‐1β‐induced nitric oxide and MMP13 production	[200]
Infrapatellar fat pad MSCs	Exosome isolation	OA mouse	Enhance mTOR mediated autophagy by miR‐100‐5p	[185]
Mouse BM‐MSCs	Exosome isolation	CIA mouse	Inhibited T cell and B cell activity	[201]
Mouse BM‐MSCs	Exosome isolation	Myocardial I/R mouse	miR‐182 promotes M2 polarization	[202]
Mouse BM‐MSCs	Exosome isolation	Myocardial I/R mouse and swine	miR‐125a‐5p promote M2 polarization, and inhibits fibroblast proliferation and activation	[203]
Human UC‐MSCs	Small EV isolation	OA mouse	M2 polarization of macrophage is promoted by STAT1 pathway	[204]
Porcine cardiac adipose tissue‐derived MSCs	Acellular cardiac scaffolds	Acute MI pig model	Treated sites have reduced inflammation‐related cytokine level	[205]
Mouse BM‐MSCs	Iron oxide‐PLGA nanoparticles to activate autophagy	NA	Enhanced autophagy promotes the EV production of MSCs	[206]
Human embryonic stem MSCs (hES‐MSCs)	MYC immortalization	NA	Conserved immunomodulatory exosome functions during large scale production	[207]
Human infrapatellar fat pad MSCs	TNF‐α preconditioning	OA mouse	Induced PI3K/AKT pathway elevate ATG16L1‐dependent autophagy and result in exosome production	[14]
Mouse hair follicle MSCs	Hypoxia‐preconditioned exosomes	Colitis mouse	miR‐214‐3p suppressed PI3K/AKT/mTOR pathway and promoted mitochondria activity	[208]
Human subcutaneous fat MSCs	Chondrocyte‐binding peptide‐modified exosomes	OA rat	Enhance mTOR mediated autophagy by miR‐199‐3p	[31]
Human UC‐MSCs	HSTP peptide‐modified exosomes	Liver fibrosis rat	Enhanced targeting HSC‐T6 cells of EV via surface modification	[209]
Not made public	CD47‐decorated exosomes	Myocardial I/R mouse	Decorated CD47 facilitates the EV to evade phagocyte clearance; miR‐21 help reduce local inflammation	[47]
BM‐MSCs	Curcumin pretreatment	OA mouse	Rescued miR‐143 and miR‐124 expression suppresses development of OA	[210]
Human UC‐MSCs	3D culture of MSCs in bioreactor	Acute kidney injury mouse	Much higher production of exosomes while ability of suppressing T cell and macrophage infiltration is maintained	[211]
Human AD‐MSCs	IFN‐γ and TNF‐α pretreatment	Temporomandibular joint rabbit	miR‐27b‐3p promotes M2 polarization by CSF‐1	[212]
Rat BM‐MSCs	Small EV isolation	SCI rat	M2 polarization of macrophage is promoted	[213]

Exosomes derived from BM‐MSCs alleviate GVHD in mouse hematopoietic stem cell transplantation (HSCT) models. These exosomes inhibited the expression of costimulatory molecules and cytokine secretion in dendritic cells, suppressed T lymphocyte proliferation, and promoted M2 macrophage polarization. In a published in vivo mouse study, these exosomes enhanced Treg cell differentiation, reduced GVHD scores, and improved survival rates [196]. Similarly, in the chronic GVHD (cGVHD) mouse model induced by injection of bone marrow cells and spleen cells, exosomes derived from BM‐MSCs effectively prolonged survival and reduced clinical and pathological scores. They inhibited the activation and infiltration of CD4+ T cells, suppressed IL‐17‐expressing pathogenic T cells, and induced IL‐10‐expressing regulatory T cells [197]. Exosomes from other MSC sources also demonstrate therapeutic effects. For instance, exosomes from human placental MSCs have been shown to inhibit T helper 17 cells cell differentiation through IκBζ downregulation, alleviating liver fibrosis in mice and human organoids [198]. miR‐27b‐3p, identified in exosomes from hUC‐MSCs, suppressed Yes‐associated protein (YAP)/ lysyl oxidase‐like 2 (LOXL2) pathway, alleviating fibrosis in carbon tetrachloride (CCl4)‐induced mouse models [184]. hUC‐MSC exosomes enhance antioxidant defenses and modulate the Nrf2 pathway, reducing oxidative stress‐related inflammation and promoting regeneration in mouse skin injury models [199]. In arthritis treatment, exosomes derived from immortalized hES‐MSCs counteracted IL‐1β effects, reducing pain and inflammation while improving matrix performance in temporomandibular joint OA rat models [200]. Infrapatellar fat pad MSC exosomes containing miR‐100‐5p inhibit the mammalian target of rapamycin (mTOR) pathway, enhancing chondrocyte autophagy and maintaining cartilage homeostasis in OA models [185]. Mouse BM‐MSCs‐derived exosomes also shifted immune responses from Th1‐type to Th2‐type in collagen‐induced arthritis (CIA) mouse models [201]. In SCI rats, three injections of BM‐MSCs‐derived small EVs were reported to upregulate TGF‐β expression of local cells, which could induce local tissue repair and increase M2 macrophage polarization [213]. Additionally, in mouse myocardial ischemia models, miR‐182 and miR‐125a‐5p within BM‐MSC exosomes promote M2 macrophage polarization, creating a favorable environment for heart regeneration [202, 203]. Currently, small EVs from hUC‐MSCs, proven to polarize macrophages to M2 states, have been applied in clinical trials for OA treatment and have shown no adverse effects [204].

However, in spite of the profound potential of exosomes derived from MSCs, there are still several limitations that hinder their clinical application. One major challenge is the limited secretion of exosomes from MSCs, which restricts their production and application efficiency [214]. In addition, the inherent heterogeneity and uncontrolled biological functions of MSC‐derived exosomes pose significant obstacles to their clinical use [119]. The stability and durability of exosomes in vivo are also concerns, as they have a short lifespan and low stability in therapeutic settings, which limit their effectiveness in applications [215]. Furthermore, the standardization of exosome isolation, dosage, and delivery methods remains a significant challenge, complicating their translation into clinical therapies [98].

To address these challenges, a range of engineering strategies is currently being implemented to modulate the EVs and exosomes derived from MSCs. These approaches aim to further augment their immunomodulatory functions and enhance their potential for inflammatory intervention and improve their therapeutic efficacy in clinical applications.

### 5.1 EV/Exosome Preconditioning and Optimized Preparation of EVs/Exosomes

Preconditioning exosome production through exposure of cells to biological, chemical, and physical factors is an effective strategy for enhancing immunomodulatory potency. For biological and chemical factor preconditioning, research has demonstrated that lipopolysaccharide (LPS)‐preconditioned rat BM‐MSCs‐derived exosomes exhibited significant anti‐inflammatory effects and improved graft survival, primarily through the promotion of M2 polarization, suppression of M1 polarization, and reduction of NF‐κB/NLRP3 signaling pathway activation [188]. TNF‐α is frequently utilized to simulate inflammatory environments. Exosomes from TNF‐α‐treated human gingiva‐derived MSCs (GMSCs) enhance M2 polarization and cartilage recovery, with miR‐1260b identified as a key mediator in murine models [15]. Similarly, TNF‐α‐treated human Gingiva‐derived MSC (G‐MSC)‐derived exosomes exhibited immunomodulatory functions in retinal models by regulating TNF‐α and IL‐1β levels in mice, protecting the retina from further tissue loss caused by inflammation [16]. MSCs acquired from infrapatellar fat pad preconditioned with TNF‐α demonstrated increased exosome production mediated by increased autophagy‐related protein 16 like 1 (ATG16L1) level, which can rescue OA cartilage [14]. Both EVs from AD‐MSCs with or without IFN‐γ and TNF‐α stimulation showed regeneration‐promoting ability in the temporomandibular joint, while EVs from stimulated cells exhibited higher immunosuppressive capability. It is suggested that it is also miR‐27b‐3p making the difference, which targets macrophage colony‐stimulating factor‐1 (CSF‐1) [212]. Fucoidan‐preconditioned rat BM‐MSCs yield exosomes with anti‐inflammatory properties and the ability to rescue the ECM, leveraging miR‐146b‐5p to protect cartilage tissue while promoting chondrocyte autophagy [189]. Similarly, pre‐treating BM‐MSCs with curcumin elevated the secretion of miR‐143 and miR‐124 via exosomes, regulating the miR‐124/NF‐κB and miR‐143/ Rho associated coiled‐coil containing protein kinase 1 (ROCK1)/Toll like receptor 9 (TLR9) pathway to protect cartilage cells from OA [210].

In terms of physical factor preconditioning, commonly employed methods include hypoxia, ultrasound, and electric stimulation. Hypoxia‐preconditioned hair follicle MSCs produced exosomes containing miR‐214‐3p, significantly reducing oxidative stress responses and protecting the digestive tract from colitis via suppression of the PI3K/AKT/mTOR pathway [208]. EVs derived from hypoxia‐preconditioned human olfactory mucosa MSCs significantly enhance angiogenesis in mice compared to those from normoxic conditions. The study identifies miR‐612 as a crucial mediator in this process, as its higher abundance in hypoxic EVs correlates with increased angiogenic activity [216]. BM‐MSCs exposed to low‐intensity pulsed ultrasound (LIPUS) yield higher anti‐inflammatory EV production where higher expression of miR‐328‐5p and miR‐487b‐3p are found. The up‐regulated miRNA can inhibit inflammation via the MAPK pathway [191]. By employing electric stimulation, exosome secretion of UC‐MSCs can be significantly elevated, benefitting exosome production [217].

Notably, You et al. employed tangential flow filtration for exosome isolation, along with click‐chemistry‐based surface modification, enhancing their affinity for macrophages. The method allowed for a 90% reduction in the required dosage to achieve comparable therapeutic outcomes in the rheumatoid arthritis mouse model compared to unmodified exosomes [190]. These findings highlight preconditioning as a versatile approach for optimizing exosome functionality across therapeutic contexts.

### 5.2 Combining EVs/Exosomes with Biomaterials

The integration of exosomes with biomaterials significantly enhances their controlled release and amplifies their immunomodulatory and anti‐inflammatory properties. For instance, Bao et al. encapsulated mouse BM‐MSC‐derived exosomes in porous PLGA microcapsules (MExoCap), achieving controlled release of >95% of exosomes over five weeks. A single intravitreal injection (5 µg exosomes) significantly promoted retinal recovery in a mouse model of retinal ischemia‐reperfusion injury (RIRI), restoring function to near‐healthy levels [192]. Han et al. proposed an exosome‐hydrogel hybrid microneedle array patch to achieve sustainable in situ exosome release, aiming to avoid secondary injury in SCI patients. The exosomes used were obtained from 3D cultured MSCs to enhance immunomodulation and regeneration‐promoting effects, validated by SCI rat models [193]. 3D culture of human UC‐MSC in a hydrophilic‐polysulfone hollow fiber bioreactor significantly enhanced exosome yield, producing up to 19.4‐fold more exosomes compared to 2D culture. Meanwhile, the efficacy of the exosome was also promoted, as evidenced by reduced inflammatory factors, repressed T cell and macrophage infiltration, improved renal function, and reduced kidney damage in a murine model of cisplatin‐induced acute kidney injury (AKI) [211]. Chen et al. utilized hBM‐MSC exosomes within a radially oriented ECM bioink, fabricated via desktop‐stereolithography 3D printing. According to the results obtained from in vitro and cartilage defect rabbit model, the biomaterial can rescue mitochondrial dysfunction and promote M2 macrophage polarization, while facilitating local chondrocyte migration [194]. Furthermore, acellular cardiac scaffolds loaded with EVs modulated systemic immune responses in a porcine myocardial infarction model, reducing TNF‐α, increasing IL‐1RA, and decreasing macrophage/T cell infiltration [195, 205].

These advancements illustrate the transformative potential of engineered materials in optimizing the therapeutic applications of exosomes across diverse biomedical fields.

### 5.3 Modification of MSCs and EVs/Exosomes

Modifying MSCs or their derived EVs / exosomes presents a viable strategy for regulating immune responses associated with EVs. For instance, hES‐MSCs can be immortalized through V‐myc avian myelocytomatosis viral oncogene homolog (MYC) transformation. Exosomes derived from these immortalized cells retain conserved immunomodulatory functions comparable to those from untreated MSCs [207]. By embedding chondrocyte‐binding peptides on the surface of exosomes, these exosomes can be effectively delivered to deep cartilage tissue to reduce OA [31]. Similarly, modifying human umbilical cord MSC‐derived exosomes with HSTP, a peptide targeting HSC‐T6 cells, significantly enhanced their therapeutic potential in treating liver fibrosis [209]. Additionally, decorating exosomes with CD47 on their surface has been shown to impede clearance by the mononuclear phagocyte system, which typically limits effective delivery. This modification results in notable reductions in apoptosis and inflammation in hearts treated with CD47‐expressing EVs in vivo [47]. Furthermore, a type of novel bioavailable nanoparticles (NPs) based on iron oxide with PLGA was recently invented to be introduced to MSCs. The NP can activate autophagy‐related factors of mice BM‐MSCs and consequently promote exosome secretion with more anti‐inflammatory factors [206].

However, despite their significant therapeutic potential, EV‐based therapies face substantial hurdles other than therapeutic efficacy. Key challenges include the unguaranteed quality, low production, and unverified storage stability [214]. Addressing these barriers in the process of production will be essential to unlock the full promise of EVs as next‐generation therapeutics. Progress has been made to overcome these hurdles. For instance, photosensitive nanoprobes are invented to isolate EV with higher purity compared to ultracentrifugation [218]. Meanwhile, various approaches for 3‐dimensional exosome production have been explored for mass production of EVs [109]. As the limitations associated with EVs continue to be addressed, MSC‐derived EVs are expected to significantly enhance the clinical applications of MSCs.

## 6 Current Research Development on iPSC‐Derived MSCs

Pluripotent stem cells, including ESCs and iPSCs, can be derived from blastocyst‐stage embryos or by reprogramming somatic cells. These cells possess the ability to differentiate into almost all types of somatic cells. Benefiting from the outstanding differentiation potency of the cells, ESCs and iPSCs have gained attention for applications in tissue regeneration [219, 220, 221].

iPSCs are generated from somatic cells through the overexpression of the Yamanaka Factors, which reprogram these cells to an ESC‐like state, endowing them with high potency [222]. In the past two decades, additional methods based on different techniques have been developed to generate iPSCs, including but not limited to viral‐vector‐based reprogramming, transposon‐based reprogramming, plasmid‐based reprogramming, and small‐molecule‐based chemical reprogramming [220, 223, 224]. Regardless of the generation method, iPSC‐derived mesenchymal stromal cells (iMSCs) offer a promising solution to address the significant challenge of MSC heterogeneity in the clinical application of MSCs [225].

Traditional MSCs exhibit considerable variability due to differences in tissue origin, donor characteristics, and age, which affect their transcriptomes, proteomes, and epigenomes, leading to inconsistent therapeutic outcomes [226, 227]. iMSCs, however, are generated from iPSCs, which can be derived from a single cell source, allowing for the pre‐selection of immunomodulatory‐cell clones and providing a more standardized and potentially unlimited supply of MSCs. This process helps to mitigate the heterogeneity associated with primary MSCs by yielding a more uniform cell population [228, 229]. During the differentiation of iPSCs into MSCs, epigenetic and chromatin remodeling occur, resulting in a rejuvenated gene expression pattern that overcomes age‐related limitations such as reduced differentiation potential and proliferation capacity [226]. The clonal dominance observed in iMSCs, derived from a limited number of individual iPSCs, further contributes to their reduced heterogeneity compared to primary MSCs, which exhibit more pronounced subclonal diversity during culture expansion [230].

iMSCs are proven to be effective in tissue repair. When implanting iMSCs into the mouse vitreous cavity, iMSCs were found to maintain retinal ganglion cells at the injected site in mice with retinal degeneration [231]. In addition, iMSCs are also applicable in the cell‐enhanced approaches mentioned in the previous sections. Exosomes obtained from iMSCs can be decorated with bone‐targeting peptide to enhance osteoporosis treatment and promote angiogenesis [232]. iMSCs‐derived exosomes have shown improved enhanced therapeutic efficacy in mouse cardiomyopathy treatment compared to BM‐MSCs‐derived exosomes, primarily by inhibiting local cell senescence [233]. Moreover, scaffold‐seeded iMSCs have shown efficacy in tissue repair; for instance, a sodium alginate hydrogel carrying iMSCs not only facilitated the histomorphological recovery in endometrial damage, but also promoted the in situ tissue cell proliferation in rat endometrial injury models [234].

Despite their potential, iMSC phenotypes vary depending on their cell source lineage. For example, it was observed that iMSCs derived from iPSCs obtained by reprogramming MSCs exhibit different functional characteristics compared to the original MSCs from the same donor [225]. Differences in immunomodulation and exosome production have been observed between iMSCs derived from different somatic cells [235]. Further research is needed to explore the performance of various iMSC subtypes to fully exploit their therapeutic potential. Meanwhile, protocols for inducing iMSCs vary across studies [110]. Whether this variability results in altered iMSC performance remains to be determined.

## 7 Discussion and Future Direction

MSCs can be isolated from a number of tissues, endowing them with high accessibility. MSCs are identified in adipose tissue, bone marrow, umbilical cord, Wharton's jelly, cord blood, placenta, dental pulp, bone, muscle, and other resources [236]. In this review, we have discussed the engineering approaches applied to different MSCs. UC‐MSCs, AD‐MSCs, and BM‐MSCs are the most predominantly studied cells in the cases discussed (Tables 1, 2, 4, 5, 6). However, the biological properties of MSCs differ significantly depending on their tissue of origin [237]. For MSC‐based applications, the source of MSC is crucial. Despite sharing a common nomenclature, MSCs derived from distinct sources exhibit different phenotypes and varying differentiation capacities. On the basis of scRNA‐seq analysis, MSCs acquired from UC, synovial tissue, BM, and AD shared similar MSC sub‐groups with different abundance [238]. Compared to UC‐MSCs, BM‐MSCs are more capable of bone and cartilage and bone differentiation, with a relatively lower potential of adipose differentiation [238, 239]. It is suggested that, in cartilage repair, BM‐MSCs are more preferable due to their low immunogenicity and decent clinical performance [240, 241]. ECM production is one of the desired biological functions of MSCs in tissue regeneration. It is reported that MSCs acquired from human bone marrow and human adipose tissue synthesize ECM with distinct compositions, specifically the expression levels of fibronectin, and collagen types I and III [197]. Conversely, in the context of neurogenesis, the differences are minimal between BM‐, AT‐, and WJ‐MSCs, as all exhibit comparable efficacy in stimulating dorsal root ganglion neurite outgrowth and protecting neural stem/progenitor cells from H_2_O_2_‐induced damage [198]. Meanwhile, the migration capacity of MSC, which is also crucial in tissue repair, varies as well, among BM‐, AT‐, UC‐, gingiva derived (G)‐, and placanta derived (P)‐MSCs [199, 200, 201]. Although immunomodulatory capability is a conserved feature across different MSCs, specific immune properties are source‐dependent. Upon culture in stimulated inflammation milieu, AD‐MSC, WJ‐MSC, and BM‐MSC showed varying phenotypes in terms of cytokine expression profiles, such as VEGF, IDO, and PTGS‐2, and others [24]. Therefore, when employing MSCs for the treatment of immune diseases and tissue regeneration, it is imperative to thoroughly evaluate the distinct characteristics of MSCs derived from various tissue sources and to select the most appropriate type and cell‐line for each specific application.

The immunomodulatory properties of MSCs have been extensively validated, highlighting their significant potential in treating inflammation‐related diseases, promoting local tissue regeneration, and protecting the tissues from further inflammatory damage through MSC‐based therapies that leverage their potent immune‐suppressive capabilities. While the prospects for the clinical application of MSC‐based therapies appear optimistic, concerns exist regarding the limited progression of trials to Phase III and Phase IV, and the scarcity of recent regulatory approvals, as discussed earlier in Section 2.

The lack of a clear and consensus‐based definition for MSCs, along with the under‐reporting of critical parameters in MSC clinical studies, significantly hinders their clinical application [242]. In this review, the cells discussed in most cases are considered as “Mesenchymal Stem Cells” as they are identified by their trilineage differentiation property. However, debate persists among scientists about the definition of MSCs. In 2006, the International Society for Cell & Gene Therapy (ISCT) identified MSCs based on positive expression of CD73, CD90, and CD105, and negative expression of hematopoietic and endothelial markers such as CD11b, CD14, and HLA‐DR. Reflecting the evolving understanding of MSCs within the research community [243], the ISCT published an updated standard for defining MSCs in 2025. This update stipulates that “Mesenchymal Stromal Cells” is the preferred term for MSCs, and mesenchymal stromal cells and mesenchymal stem cells are no longer interchangeable terms. The standard maintains the basic requirement for positive markers (CD73, CD90, and CD105) and the negative marker CD45, while specifying that in vitro trilineage differentiation is no longer mandatory for MSC identification [242]. In 2025, the Chinese drug approval authorities have started a discussion regarding the terms used for MSCs in the regulatory documents, suggesting to use “Mesenchymal Cells” instead of “Mesenchymal Stem Cells” or “Mesenchymal Stromal Cells”, followed by a case‐by‐case approval procedure, thus to expedite the regulatory review process [244]. Another key aspect of the new standard emphasizes that detailed information on MSCs used in clinical applications must be published, including specific details such as cell source, culture conditions, preservation methods, and donor information, owing to the inherent heterogeneity of MSCs [242]. Notably, during the preparation of this review, collecting such detailed information from clinical trials has proven to be challenging. Data specified in the new consensus, such as donor details or preservation methods, are frequently unavailable (e.g., listed as NA in Tables 1 and 2). The lack of publicly accessible information undermines the credibility and reproducibility of reported trials for scientists and clinicians, and may also impede regulatory approval. Consensus in implementing this new standard could bring a more unified requirement to the field, and enhance the transparency, manageability, and reproducibility of both pre‐clinical and clinical research.

Substantial evidence presented in this article indicates that reducing inflammatory responses promotes tissue regeneration. However, it is critical to emphasize that maintaining a dynamic balance between inflammation suppression and enhancement is essential for optimal tissue regeneration, rather than solely excluding inflammation. Inflammation serves as an immediate response to injury, initiating the clearance of damaged cells and pathogens [245]. The dual function of inflammation, protective and regeneration‐stimulating, is pivotal in initiating tissue regeneration during the very early stage of tissue wounds [246]. For instance, in the lungs, inflammation not only protects against pathogens but also stimulates lung‐resident stem cells to promote tissue regeneration, highlighting its role in both defense and repair mechanisms [247]. In the context of MSC‐mediated regeneration, inflammatory factors mediate MSC recruitment, while enhancing MSC differentiation and migration, consequently promoting regeneration [248]. In this review, we summarize bioengineering strategies that have emerged as a potential solution to enhance the functionality and applicability of MSCs. Techniques such as genetic modification, hypoxic preconditioning, and the use of biomaterials were investigated, aiming to improve cell viability, enhance immunomodulatory effects and regenerative capacity, and optimize delivery methods (Tables 4, 5, 6 and Figures 2, 4, and 5), supporting the future clinical applications of MSC‐based therapies.

In conclusion, the exploration of MSC‐based therapy in immunoregulation presents a compelling avenue to enhance its applications in immunomodulation and tissue regeneration. This review has highlighted significant advancements in the field, particularly from the point of view of Phase III and IV clinical trials and regulatory approvals, which underscore the therapeutic promise of MSCs. However, the journey toward clinical application is fraught with challenges. By leveraging innovative engineering strategies, the consistency and efficacy of MSC therapies may be significantly enhanced. Besides, while scientists are building up a more unified standard for MSC‐therapy, the regulatory agencies are alsoadvancing the clinical translation forward, accelerating the approval and providing a more supportive environment [249, 250]. The future of MSC‐based therapy is optimistic. Coupled with rapid advances in bioengineering, these converging scientific and regulatory trends signal a turning point for the field. As challenges related to scalability, consistency, and mechanistic clarity continue to be addressed, the therapeutic potential of MSCs is increasingly being realized in real‐world clinical settings. Consequently, the future of MSC‐based therapy is not only optimistic—it is poised for transformative impact across a broad spectrum of unmet medical needs, from autoimmune and inflammatory diseases to tissue regeneration and beyond.

## Funding

The National Key R&D Program (Grant No. 2019YFA0111900; Y.J.), administered by the Ministry of Science and Technology of the People's Republic of China (MOST, China); NSFC/RGC Joint Research Scheme sponsored by the Research Grants Council of the Hong Kong Special Administrative Region, China and the National Natural Science Foundation of China (Project No. _CUHK483/22; Y.J.); Health@InnoHK, Innovation and Technology Commission, the Government of the Hong Kong Special Administrative Region of the People's Republic of China (Centre for Neuromusculoskeletal Restorative Medicine; Y.J. and R.S.T.); Guangdong Basic and Applied Basic Research Foundation (No. 2023B1515130006); Regulatory Science Research Project of the Greater Bay Area Sub‐Center for Drug Evaluation and Inspection of National Medical Products Administration (No. GBA‐JGKX‐2404); The Lee Quo Wei and Lee Yick Hoi Lun Professorship in Tissue Engineering and Regenerative Medicine of the Chinese University of Hong Kong (R. S. T.).

## Conflicts of Interest

The authors declare no conflicts of interest.
